# IEV2Mol: Molecular Generative Model Considering Protein–Ligand Interaction Energy Vectors

**DOI:** 10.1021/acs.jcim.4c00842

**Published:** 2024-09-10

**Authors:** Mami Ozawa, Shogo Nakamura, Nobuaki Yasuo, Masakazu Sekijima

**Affiliations:** † Department of Computer Science, Tokyo Institute of Technology, Yokohama, Kanagawa 226-8501, Japan; ‡ Department of Life Science and Technology, Tokyo Institute of Technology, Yokohama, Kanagawa 226-8501, Japan; § Academy for Convergence of Materials and Informatics (TAC-MI), Tokyo Institute of Technology, Tokyo 152-8550, Japan

## Abstract

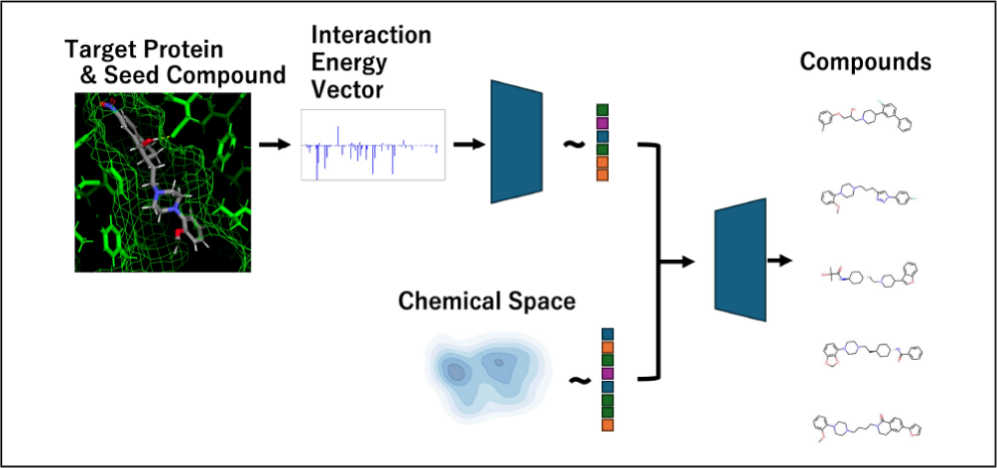

Generating drug candidates with desired protein–ligand interactions is a significant challenge in structure-based drug design. In this study, a new generative model, IEV2Mol, is proposed that incorporates interaction energy vectors (IEVs) between proteins and ligands obtained from docking simulations, which quantitatively capture the strength of each interaction type, such as hydrogen bonds, electrostatic interactions, and van der Waals forces. By integrating this IEV into an end-to-end variational autoencoder (VAE) framework that learns the chemical space from SMILES and minimizes the reconstruction error of the SMILES, the model can more accurately generate compounds with the desired interactions. To evaluate the effectiveness of IEV2Mol, we performed benchmark comparisons with randomly selected compounds, unconstrained VAE models (JT-VAE), and compounds generated by RNN models based on interaction fingerprints (IFP-RNN). The results show that the compounds generated by IEV2Mol retain a significantly greater percentage of the binding mode of the query structure than those of the other methods. Furthermore, IEV2Mol was able to generate compounds with interactions similar to those of the input compounds, regardless of structural similarity. The source code and trained models for IEV2Mol, JT-VAE, and IFP-RNN designed for generating compounds active against the DRD2, AA2AR, and AKT1, as well as the data sets (DM-QP-1M, active compounds to each protein, and ChEMBL33) utilized in this study, are released under the MIT License and available at https://github.com/sekijima-lab/IEV2Mol.

## Introduction

Structure-based drug discovery (SBDD) plays a critical role in drug development.^1−4^ SBDD is a method that uses the three-dimensional structure of target proteins to optimize their interactions with ligands and to rationally design new drug candidates to simultaneously meet a wide range of optimization goals, such as activity, selectivity, and physical properties.^5,6^

SBDD has been successfully applied in many drug discovery projects.^7^ For example, in the development of celecoxib, a selective COX-2 inhibitor,^8^ structure–activity relationship studies of 1,5-diarylpyrazole derivatives focused on optimizing physical properties while maintaining COX-2 inhibitory activity and selectivity, ultimately leading to the discovery of a compound with high inhibitory activity and selectivity.^9^

In the development of therapeutics targeting the 3CL protease of SARS-CoV-2 in response to the global COVID-19 pandemic,^10,11^ docking-based virtual and biological screening using the SBDD approach identified several hits with IC_50_ values below 10 μM.^12^ One of the hits, compound 1, was selected for clinical development. Compound 1 was structurally optimized by SBDD using X-ray cocrystal structures, resulting in a more than 600-fold increase in activity, ultimately yielding S-217622, a nonpeptidic, noncovalently bound oral 3CLpro inhibitor.

Docking simulations using existing libraries and the resulting in vitro and in vivo assays have successfully identified promising hits in many studies.^13−16^ However, the best compounds are not always included in the library. To achieve the ultimate goal of drug discovery, i.e., to discover novel drug candidates with desirable pharmacological properties and few side effects, innovative methods that can efficiently explore the vast space of 10^6017^ chemical compounds and generate highly relevant compounds are needed.

In recent years, with the rapid development of artificial intelligence (AI) technology, its application to molecular design has attracted considerable attention. In particular, deep learning models such as recurrent neural networks (RNNs),^18−20^ variational autoencoders (VAEs),^21,22^ generative adversarial networks (GANs),^23−25^ and graph neural networks (GNNs)^26^ have successfully generated novel compounds using molecular graphs and simplified molecular input line entry system (SMILES)^27^ representations and are expected to be powerful tools for molecular design.

These models can learn a distribution of chemical structures from a large compound database and generate new structures. A ligand generation method that combines transfer learning and docking score optimization has been proposed to design new ligands by using deep learning to exploit the pocket information on target proteins, even in the absence of known ligand information for the target protein, by using the ligand information on proteins belonging to the same family.^28^ On the other hand, there are many cases where the interaction of a specific residue with a ligand should be considered.

A conditional recurrent neural network (cRNN) model utilizing ligand/protein interaction fingerprinting (IFP)^29−33^ has been proposed to generate compounds that interact with target proteins.^34^ IFP is a binary vector based on the docking pose that is automatically constructed to indicate whether a ligand interacts with a protein, and incorporating this vector into the cRNN model enables the generation of novel ligand structures with the desired binding mode for a specific target. However, the IFP only considers the presence or absence of interactions and does not reflect the strength of the interaction. Virtual screening has already shown that considering the strength of interactions is more accurate than considering the IFP alone.^35^

In this study, we introduce the interaction energy vector (IEV) as a descriptor that quantitatively measures the strength of the interaction between a protein and a ligand. The IEV is obtained from docking simulations. It is calculated for each type of interaction, including hydrogen bonding, electrostatic interactions, and van der Waals forces.^35^ This vector provides a comprehensive representation of the protein–ligand interaction landscape, consisting of elements corresponding to each interaction type. This IEV is used as input to a variational autoencoder (VAE) model, which is trained end-to-end with another VAE that learns the chemical space from SMILES representations. By minimizing the reconstruction error of the SMILES in the joint latent space during training, the model learns to generate compounds with the desired interactions more accurately.

To evaluate the effectiveness of the proposed method, we performed benchmark comparisons with randomly selected compounds, the unconstrained VAE model (JT-VAE), and compounds generated by the IFP-RNN. The results confirmed that the compounds generated by the proposed method have a significantly greater rate of maintaining the binding mode of the query structure. These results indicate that IEV2Mol has the potential to be a useful tool for the generation of novel compounds with desirable protein–ligand interactions, and it is expected to contribute to the efficiency of the drug discovery process.

## Materials and Methods

### Docking Simulation

This research used docking simulations in Glide SP mode to calculate the interactions between the target protein and the generated compounds.^36^ The protein preparation wizard was used for hydrogen addition and structural minimization. LigPrep was used to generate ligand tautomeric and ionization states, ring conformations, and stereoisomers at pH 7.4. Further analysis was conducted using the best-scored pose.

### Construction of the Interaction Energy Vector

The interaction fingerprint is used to quantitatively calculate the similarity of protein–ligand interactions. It is a bit-string representation based on the presence or absence of interactions. IEV (interaction energy vector) is a method inspired by the interaction fingerprint and was developed by Yasuo and Sekijima.^35^ This method differs from the interaction fingerprint in that the IEV is a vector of real-valued protein–ligand interactions based on energy, whereas the interaction fingerprint is a sequence of bits expressed as 0/1 values depending only on the presence of an interaction. The IEV is calculated using the following procedure. First, for each amino acid residue within 12 Å of the center of the docking grid, van der Waals forces, Coulomb forces, and hydrogen bond energy values are calculated for all atomic pairs between the docked compound and the residue. The three interaction energy values for each amino acid residue are then calculated by summing the energy terms of the atoms within each amino acid residue. The van der Waals forces, Coulomb forces and hydrogen bonds are then ranked in order of the number assigned to each amino acid residue in the target protein’s PDB file. In other words, the length of the IEV vector is three times the number of amino acid residues within 12 Å of the center of the docking grid, which varies for each target protein and is therefore a unique representation for each protein. These calculations can be performed in docking simulations using Glide.^36,37^

### Data Sets

In the present study, to prevent the model from overfitting to the chemical space of active compounds and to learn a broad chemical space, the DM-QP-1 M data set was used.

In addition, dopamine receptor D2 (DRD2), adenosine receptor A2 (AA2AR), and AKT serine/threonine kinase 1 (AKT1) was selected as the target for evaluating the method, and the active compound data set, a data set of active compounds for each protein, was created. The DM-QP-1 M data set and active compound data set are available on GitHub.

The DM-QP-1 M data set consists of 981,139 compounds obtained by the following procedure. First, 1,000,000 compounds were randomly selected from the DM-QP data set, a data set of drug-like compounds created by Lee et al.^38^ Second, if SMILES notations were found containing multiple compounds, such as solvents, we kept only the compounds with the highest molecular weight. Finally 18,861 duplicate compounds were removed.

The active compound data set was created in this study and is a data set of compounds with activity against each protein. The active compound data set consists of 8,350, 6,640, and 3,576 compounds for DRD2, AA2AR, and AKT1 respectively, obtained by the following procedure.

First, SMILES of compounds with available *K*_i_ or IC_50_ values, which are indicators of protein binding activity, were obtained from the ChEMBL database.^39^ The active compounds for DRD2 were obtained as of September 25, 2023, while those for AA2AR and AKT1 were obtained as of June 21, 2024. Next, if there were SMILES containing multiple compounds, such as solvents, only the compound with the highest molecular weight among them was retained. Duplicate compounds were then removed. The 3D structures were then generated using LigPrep, and docking simulations with DRD2 (PDB ID: 6CM4), AA2AR (PDB ID: 3EML), and AKT1 (PDB ID: 3CQW) were performed in Glide HTVS mode to calculate the IEV. In the docking simulations, multiple docking poses could be generated for a single compound, in which case the one with the most negative docking score was selected. IEV resulting from docking to DRD2, AA2AR, and AKT1 had 189, 177, and 207 dimensions, respectively. Finally, for the ligands of each protein, the active compounds were divided into 100 clusters in the ECFP4, 1024-bit space using k-means clustering, and 100 compounds closest to the centroid of each cluster were selected to create the test data set, and the remaining compounds were selected as the training data set.

### Model Architecture

**Figure 1:**
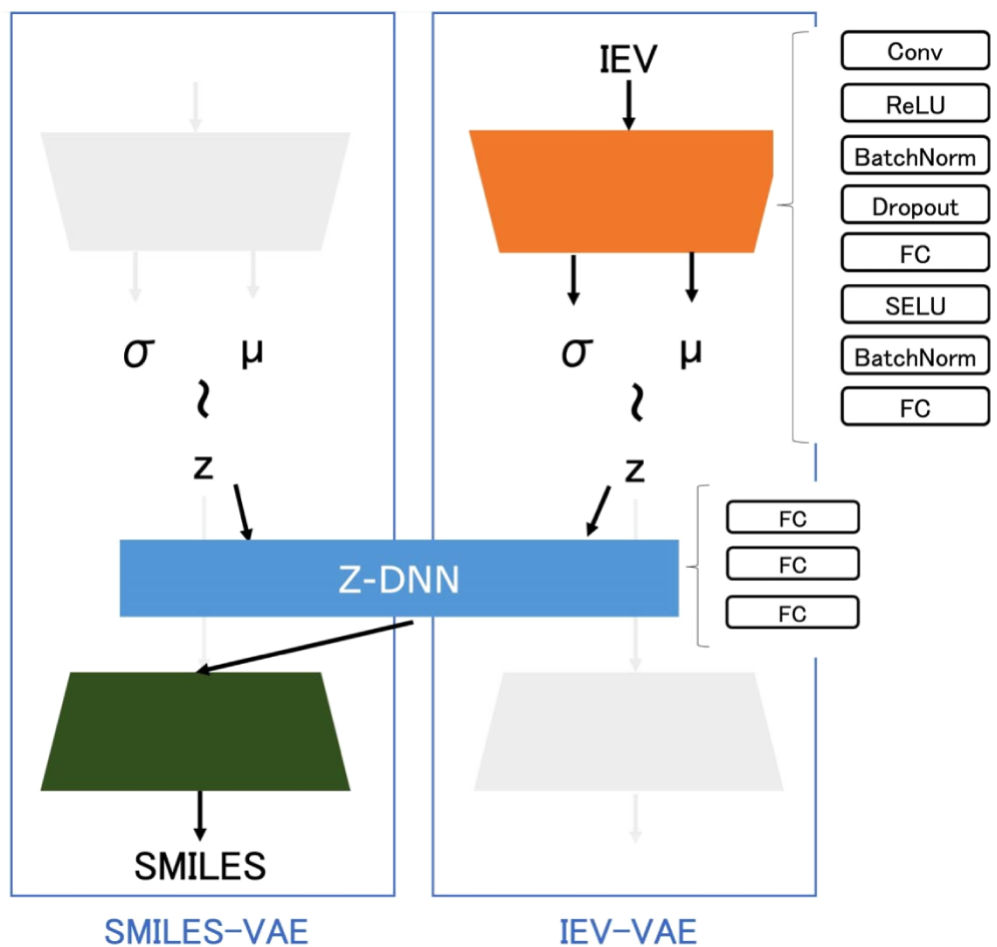
Schematic representation of the IEV2Mol framework, which integrates three key modules – SMILES-VAE, IEV-VAE, and the Z-DNN – for the generation of novel molecular compounds with desired protein–ligand interaction energy values. SMILES-VAE learns the chemical space distribution from SMILES representations, while IEV-VAE captures the interaction energy vector (IEV) distribution of active compounds. The Z-DNN combines the latent representations from both VAEs to generate new compounds with targeted interaction profiles.

The IEV2Mol architecture consists of SMILES-VAE, IEV-VAE, and Z-DNN (Figure 1).

#### SMILES-VAE

SMILES-VAE is a VAE module that uses SMILES notations as the input and output, and the VAE model of the MOSES^40^ benchmark was used. The encoder in the SMILES-VAE is composed of bidirectional GRU layers and fully connected layers, and the decoder is composed of three GRU layers with dropout. The latent space has 128 dimensions. SMILES-VAE is used for learning and using chemical space distributions.

#### IEV-VAE

IEV-VAE is a VAE module that uses the IEV as the input and output. The encoder and decoder are implemented using a combination of convolution layers (Conv) and fully connected layers (FC), along with dropout, batch normalization (BatchNorm), and activation layers, such as ReLU and SELU.

The Conv layer here is a one-dimensional convolution layer. eq 1 defines the transformed feature at the *i*th position, , as the result of the dot product between the kernel *W*_*k*_ and the input feature vector *h*_*i*_, which can be expressed as .
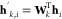
1

The BatchNorm layer is a layer that performs batch normalization, where each *i*th feature value **h**_*i*_ of the data in a batch is standardized by the operation of eq 2 so that the mean is 0 and the variance is 1 within a batch. In this case, *epsilon* = 1*e* – 5 in eq 2.
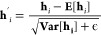
2

In the dropout layer, neurons are randomly thinned at a certain rate in each epoch during learning.

The FC layer in this study refers to the fully connected layer. Here, the input vector **h** is linearly transformed using the learned weight matrix **W** and the bias vector **b** as in eq 3.
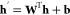
3

In the ReLU layer, the input vector **h** is activated using the ReLU activation function, as shown in eq 4.
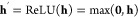
4

In the SELU layer, the input vector **h** is activated using the activation function SELU, as shown in eq 5. In this case, α = 1.6732632423543772848170429916717 and λ = 1.0507000009873554804934193349852946 in eq 5, which are the same values proposed in the original article.^41^


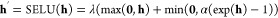
5

The latent space has 56 dimensions. IEV-VAE is employed to learn and use the IEV distribution of active compounds.

#### Z-DNN

The Z-DNN (Z-deep neural network) consists of three fully connected layers (FCs) and is designed to learn a mapping from the concatenated latent space of SMILES-VAE and IEV-VAE to the SMILES-VAE latent space. This mapping enables the generation of new compounds with the desired interaction energy profile, as represented by IEV. Since the Z-DNN has the same dimensionality as the SMILES-VAE latent space, the output of the Z-DNN can be directly fed into the SMILES-VAE decoder to generate new compounds.

### Model Construction

#### SMILES-VAE Pretraining

The DM-QP-1 M data set was used to pretrain models to learn diverse representations of a chemical space; pretraining the DM-QP-1 M data set prevents the models from overfitting the chemical space of active compounds in the target protein, allowing them to learn a wide chemical space. The SMILES-VAE was pretrained using the DM-QP-1 M data set with a batch size of 512 and the Adam optimizer for 100 epochs.

As expressed in eq 6, the loss function *L*_SMILES-VAE_ consists of a weighted sum of the reconstruction error *L*_SMILES_ due to the cross-entropy error between the correct SMILES and the probability value for each token in the predicted SMILES string, represented in eq 7, and the KL divergence (Kullback–Leibler divergence) *L*_KL_, represented in eq 8.
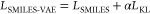
6
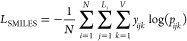
7
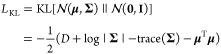
8where *N* is the batch size, *T*_*i*_ is the length of the SMILES string for the *i*th sample, *y* is the input SMILES string, *p* is the probability of output SMILES string, *V* is the size of the SMILES vocabulary. *D* is the number of dimensions of the VAE latent space, and **μ** and **Σ** are the mean and variance of the *D*-dimensional normal distribution output by the VAE, respectively. The initial value of the KL divergence weights α was set to 0 and annealed by 5e-3 per epoch. Additionally, the learning rate was fixed at 3e-4.

#### IEV-VAE Pretraining

The IEV-VAE was pretrained using the active compound training data set with a batch size of 128 and the Adam optimizer for 100 epochs in this experiment.

As expressed in eq 9, the loss function consists of the sum of the *L*_IEV-L1_, L1 loss of the correct IEV and the output IEV represented in eq 10, and the *L*_KL_, the KL divergence represented in eq 8.
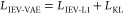
9
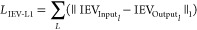
10where  is the *l*th value of the input IEV and  is the *l*th value of the output IEV. Additionally, the initial value of the learning rate was 1e-3 and was multiplied by 0.9 every 2000 batches processed.

#### End-to-End Model Training

After pretraining of the SMILES-VAE and IEV-VAE was completed, the integrated model, consisting of the pretrained SMILES-VAE, IEV-VAE, and the Z-DNN, was trained using the active compound training data set with a batch size of 128 and the Adam optimizer for 100 epochs.

During training, the SMILES and IEV pairs of the compounds in the active compound data set were used as input to the pretrained SMILES-VAE and IEV-VAE encoders, respectively. Then, the latent representations output by the two encoders were concatenated and used as input to the Z-DNN. Finally, the variables output by the Z-DNN were decoded into SMILES representations by the SMILES-VAE decoder. As expressed in eq 11, the loss function *L*_Model_ is defined by the reconstruction error of SMILES *L*_SMILES_, expressed in eq 7.
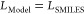
11

Note that the pretrained SMILES-VAE encoder and IEV-VAE weights are frozen, and only the SMILES-VAE decoder and Z-DNN weights are trained. The initial learning rate was 1e-4 and was multiplied by 0.8 for every 20 epochs processed.

### Compound Generation

After a series of training, IEV2Mol uses the IEV as input to generate compounds believed to have similar IEVs via the following procedure.

i The target IEV is used as input to the IEV-VAE encoder to obtain a latent representation.
ii Variables are randomly sampled from a standard normal distribution with the same number of dimensions as the SMILES-VAE latent space.
iii The variables obtained in procedures (i) and (ii) are concatenated and used as input to the Z-DNN.
iv The output of the Z-DNN is taken as input to the SMILES-VAE decoder and decoded into a SMILES representation.

### Technical Details

IEV2Mol was implemented using PyTorch^42^ along with the RDKit.^43^ The computing environment was a SUSE Linux Enterprise Server 12 SP2 with an Intel Xeon E5-2680 v4 CPU, and four NVIDIA TESLA P100 (16 GB memory) were used for the docking simulation to calculate the IEV for the active compound data set and for model evaluation. The computing environment for model training and compound generation was the Ubuntu 22.04 OS, an Intel(R) Xeon(R) Silver 4110 CPU, 64 GB of RAM, and an NVIDIA GeForce RTX 4090 GPU (with 24 GB of memory).

## Experiments and Evaluation

### Experimental Details

To evaluate IEV2Mol, we performed compound generation experiments using IEVs from the active compound test data set as input. In this experiment, 100 compounds were generated per test compound. Note that in this 100th generation, the IEV-VAE latent representation and the SMILES-VAE latent representation were newly sampled each time.

#### JT-VAE

For comparison with the proposed IEV2Mol model, a graph-based de novo generative model, JT-VAE,^44^ was trained and evaluated using the active compound training data set with a batch size of 2 and the Adam optimizer for 20 epochs. Note that we used the JT-VAE model implemented in Python3 by Bibhash Mitra, which is available from the GitHub repository https://github.com/Bibyutatsu/FastJTNNpy3.^45^

Additionally, the initial value of the learning rate was 1e-3 and was multiplied by 0.9 for every 2000 batches processed. The other settings had the default values. The models in training were saved every 10 epochs during training, and the model with the lowest validation loss was selected.

Similar to IEV2Mol, JT-VAE generated 100 compounds per test compound. Because JT-VAE does not accept IEVs as input, the SMILES of the test compounds were used as input for the generation.

#### IFP-RNN

For further comparison, the interaction fingerprint (IFP)-based cRNN generation model, IFP-RNN,^46^ was also trained and evaluated using the active compound training data set with a batch size of 500 and the Adam optimizer for 500 epochs. Note that we used the IFP-RNN model implemented in Python3 by Jie Zhang, which is available from the GitHub repository (https://github.com/jeah-z/IFP-RNN).^47^

For training, we used residue-specific IFPs calculated from the active compound data set docked to each protein in Glide HTVS mode. The learning rate was set to the default setting (the initial value was 1e-3, and after the 200th epoch, the initial value decayed through multiplication by ).

Then, similar to IEV2Mol, we generated 100 compounds per test compound in the IFP-RNN. The IFP of the test compound was used as input for the generation process.

#### Random ChEMBL

As a baseline, 100 randomly selected compounds from the ChEMBL33^48^ database, which contains approximately 2.27 million compounds, were evaluated for each test data point. Notably, 2.27 million compounds were obtained by preprocessing all SMILES sequences on ChEMBL33 to remove duplicates after removing the solvent, as was done when the data set was created.

### Metrics

The following 6 metrics were used to evaluate IEV2Mol. For comparison, we used the average values of these metrics for the compounds generated by each model using each test data set.

- Validity (number of valid compounds)
- Uniqueness (uniqueness of valid compounds)
- Diversity (diversity of valid compounds)
- Number of compounds that were able to dock to the target protein in Glide HTVS mode (number of compounds for which the IEV could be calculated)
- Number of compounds for which the cosine similarity between the IEV of the input compound and its own IEV is greater than 0.7
- Number of compounds for which the cosine similarity between the IEV of the input compound and its own IEV is greater than 0.7 and the Tanimoto coefficient with the input compound is less than 0.5

Validity, uniqueness, and diversity are metrics also used in the MOSES^40^ benchmark. Diversity was calculated by the eq 12 with *p* = 1.
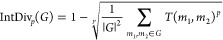
12
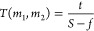
13where *G* is the set of valid compounds generated, *T* is the Tanimoto coefficient given by the eq 13, *t* is the total number of bits that are both 1 when comparing the ECFP of compound *m*_1_ with that of *m*_2_, *f* is the total number of bits that are both 0, and *S* is the number of ECFP bits. In this study, we used ECFP4 with *S* = 2048.

Among these 6 metrics, validity, uniqueness, and diversity were used to evaluate the performance of IEV2Mol as a generative model. The remaining 3 metrics were used to evaluate the ability of IEV2Mol to generate compounds that have similar interactions with the input compounds but have diverse structures, which is the objective of this study.

In addition, we plotted the distributions for the Tanimoto coefficient (ECFP4) and cosine similarity to the IEV of the seed compound for all compounds generated using all test data as input, along with the distribution of the chemical space of the data set used for training and the positions of the generated compounds. Regarding plots of the distribution of the chemical space and the location of the generated compounds, a two-dimensional plot of ECFP4 with *S* = 2048 was generated by principal component analysis (PCA) with dimensionality reduction.

Finally, we evaluated the docking poses in Glide HTVS mode for the top four compounds generated by IEV2Mol with the highest IEV cosine similarity among those with Tanimoto coefficients of 0.5 or less.

## Results and Discussion

Table 1 shows the results of evaluating the validity, uniqueness, and diversity of IEV2Mol, JT-VAE, IEV-VAE, and Random ChEMBL.

**Table 1:** Average Values of Validity, Uniqueness, and Diversity for the Compounds Generated by Each Model (IEV2Mol, JT-VAE, IFP-RNN, and Random ChEMBL) for Each Protein^a^

Target	Model	Validity	Uniqueness	Diversity
DRD2	IEV2Mol	97.5	0.987	0.835
	JT-VAE	**100.0**	0.236	0.303
	IFP-RNN	85.1	**1.000**	0.712
	Random ChEMBL	**100.0**	**1.000**	**0.880**
AA2AR	IEV2Mol	96.3	0.979	0.855
	JT-VAE	**100.0**	0.228	0.316
	IFP-RNN	74.6	**1.000**	0.755
	Random ChEMBL	**100.0**	**1.000**	**0.880**
AKT1	IEV2Mol	94.6	0.971	0.851
	JT-VAE	**100.0**	0.262	0.369
	IFP-RNN	44.7	**1.000**	0.722
	Random ChEMBL	**100.0**	**1.000**	**0.880**

a The bolded values are the best values, and the underlined values are the second-best values.

It is shown that IEV2Mol has a higher validity than the IFP-RNN, which also uses SMILES-based generation. We attribute our success to the fact that IEV2Mol was able to learn grammars on a larger data set, DM-QP-1 M data, whereas the IFP-RNN learned grammars on the active compound data set.

The IFP-RNN architecture requires the computation of IFPs for all training data, which makes it difficult to expand the training data. On the other hand, IEV2Mol required only SMILES for SMILES-VAE pretraining, making it easily scalable and allowing us to train the grammar on a larger set of DM-QP-1 M data.

Additionally, IEV2Mol was comparable to Random ChEMBL in the evaluation of uniqueness and diversity. JT-VAE yielded particularly poor results for uniqueness and diversity, which may be because JT-VAE generates compounds by sampling based on structural similarity to the input compounds. IEV2Mol, on the other hand, seems to have performed better by generating compounds through sampling based on IEV similarity.

Table 2 shows the results of the evaluation of the number of compounds that were able to dock to the target protein in Glide HTVS mode, the number of compounds for which the cosine similarity between the IEV of the input compound and its own IEV was greater than 0.7, and the number of compounds for which the cosine similarity between the IEV of the input compound and its own IEV was greater than 0.7 and the Tanimoto coefficient with the input compound was less than 0.5.

**Table 2:** Average Number of Dockable Compounds Generated by Each Model for Each Protein with IEV Cosine Similarity to the Input Compound Greater than 0.7, and Average Numbers of Generated Compounds with IEV Cosine Similarity to the Input Compound Greater than 0.7 and Tanimoto Similarity to the Input Compound Less Than 0.5^a^

Target	Model	Dockable	IEV Cos ≥0.7	IEV Cos ≥0.7 and Tanimoto ≤0.5
DRD2	IEV2Mol	91.5	27.6	**27.0**
	JT-VAE	**94.5**	**50.8**	5.1
	IFP-RNN	81.8	24.3	22.2
	Random ChEMBL	78.7	13.7	13.7
AA2AR	IEV2Mol	94.5	67.9	**66.7**
	JT-VAE	**98.8**	**77.3**	16.1
	IFP-RNN	72.2	49.4	47.2
	Random ChEMBL	79.8	38.1	38.1
AKT1	IEV2Mol	91.1	24.8	**22.7**
	JT-VAE	**96.6**	**39.8**	5.2
	IFP-RNN	39.9	7.8	6.3
	Random ChEMBL	82.3	10.8	10.8

a The bolded values are the best values, and the underlined values are the second-best values.

IEV2Mol performed better than any other case except JT-VAE in evaluating the number of generated dockable compounds and the number of generated compounds with high IEV cosine similarity. However, as discussed in Table 1, we believe that JT-VAE shows good values for these indices because it produces compounds with high structural similarity to the input compounds. Therefore, for our purposes, the JT-VAE results cannot be simply evaluated as good. This consideration is reinforced by the fact that JT-VAE showed significantly worse results when evaluated with the additional conditions of low structural similarity to the input compound as well as high IEV cosine similarity. On the other hand, IEV2Mol did not significantly weaken the results when evaluated with the additional condition of low structural similarity to the input compound. In other words, IEV2Mol succeeds in generating compounds that have similar interactions without depending on structural similarity to the input compound. These model-specific trends are consistent when targeting DRD2, AA2AR, and AKT1, showing that the ability of IEV2Mol to generate compounds with high IEV similarity and low structural similarity to seed compounds is robust across different target proteins.

**Figure 2:**
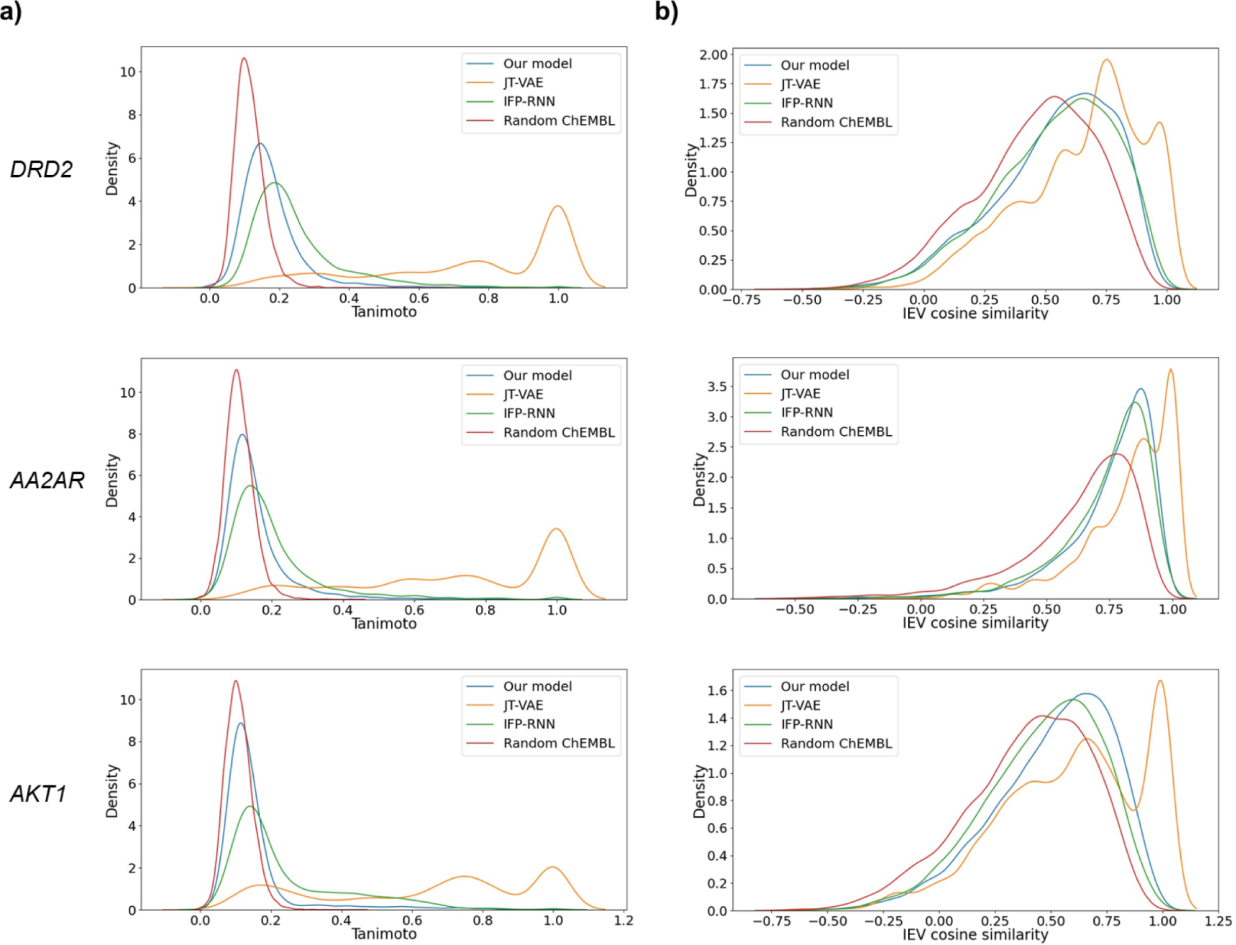
Comparative analysis of the distributions of (a) Tanimoto coefficients (calculated using ECFP4) and (b) cosine similarities of interaction energy values (IEVs) between the seed compounds and the generated compounds for each protein. The proposed IEV2Mol framework (referred to as “our model”) is compared to the JT-VAE and IFP-RNN methods, as well as to a random sample of the ChEMBL database (Random ChEMBL), with all test data used as input for each approach.

Figure 2 shows a comparative analysis of the distributions of (a) Tanimoto coefficients (calculated using ECFP4) and (b) cosine similarities of IEVs between the seed compounds and the generated compounds using kernel density estimation (KDE). The proposed IEV2Mol framework (referred to as “our model”) is compared to the JT-VAE and IFP-RNN methods, as well as to a random sample of the ChEMBL database (Random ChEMBL). All test data were used as input for each approach. The KDE plot of the Tanimoto coefficients in Figure 2a shows that IEV2Mol generates compounds with a greater degree of structural diversity than do JT-VAE and IFP-RNN, as evidenced by the greater density of compounds with lower Tanimoto coefficients. This indicates that IEV2Mol is capable of generating a diverse range of molecules that lack structural similarity to the seed compound. In terms of the KDE plot of IEV cosine similarities, shown in Figure 2b, the compounds generated by IEV2Mol show greater similarity to the seed compounds than those of IFP-RNN. This observation suggests that IEV2Mol successfully reproduces IEV even when generating compounds with no structural similarity to the seed compound. Notably, the IEV cosine similarity distribution for JT-VAE has a prominent peak at 1.0, indicating that JT-VAE tends to generate compounds with high structural similarity to the seed compound. This can be attributed to the fact that JT-VAE relies heavily on the structural information on the seed compound during the generation process.

**Figure 3:**
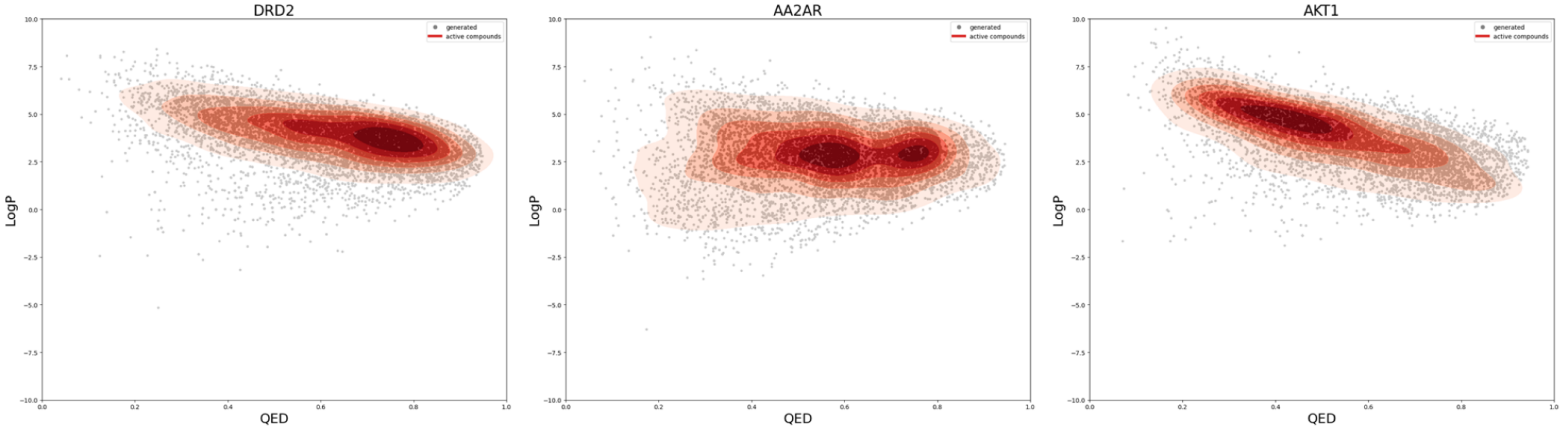
QED–LogP plots for compounds generated by IEV2Mol targeting DRD2, AA2AR, and AKT1. Points represent generated compounds, while the red density map shows the distribution of the active compounds.

Figure 3 shows the QED–LogP plots of the generated compounds for each of DRD2, AA2AR, and AKT1. These properties were calculated by RDKit. For each protein, IEV2Mol generated molecules with property distributions similar to those of active compounds, indicating that IEV2Mol learned the features of the known ligands.

Considering the objective of this study, which is to generate compounds with diverse structures while maintaining similar interaction energy profiles to the seed compound, the lower IEV cosine similarity of IEV2Mol compared to that of JT-VAE is not a problem. Indeed, it highlights the ability of IEV2Mol to generate structurally diverse compounds with interaction energy profiles similar to those of the seed compound. The results show that IEV2Mol is a promising approach for the generation of novel compounds with desired protein–ligand interactions, as it achieves a balance between structural diversity and the reproduction of interaction energy values.

Figure 4 shows that IEV2Mol generates a wide variety of compounds that cover the chemical space of the active compound, regardless of its position in the chemical space of the seed compound. Figures for all seed compounds are shown in Figures S1–S15. This result, as well as the results shown in Figure 2, suggests that IEV2Mol can generate compounds with IEVs similar to those of the seed compound, even when the generated compounds have no structural similarity to the seed compound. The ability of IEV2Mol to explore different regions of chemical space while maintaining the targeted protein–ligand interaction profile is a major advantage in the context of drug discovery.

**Figure 4:**
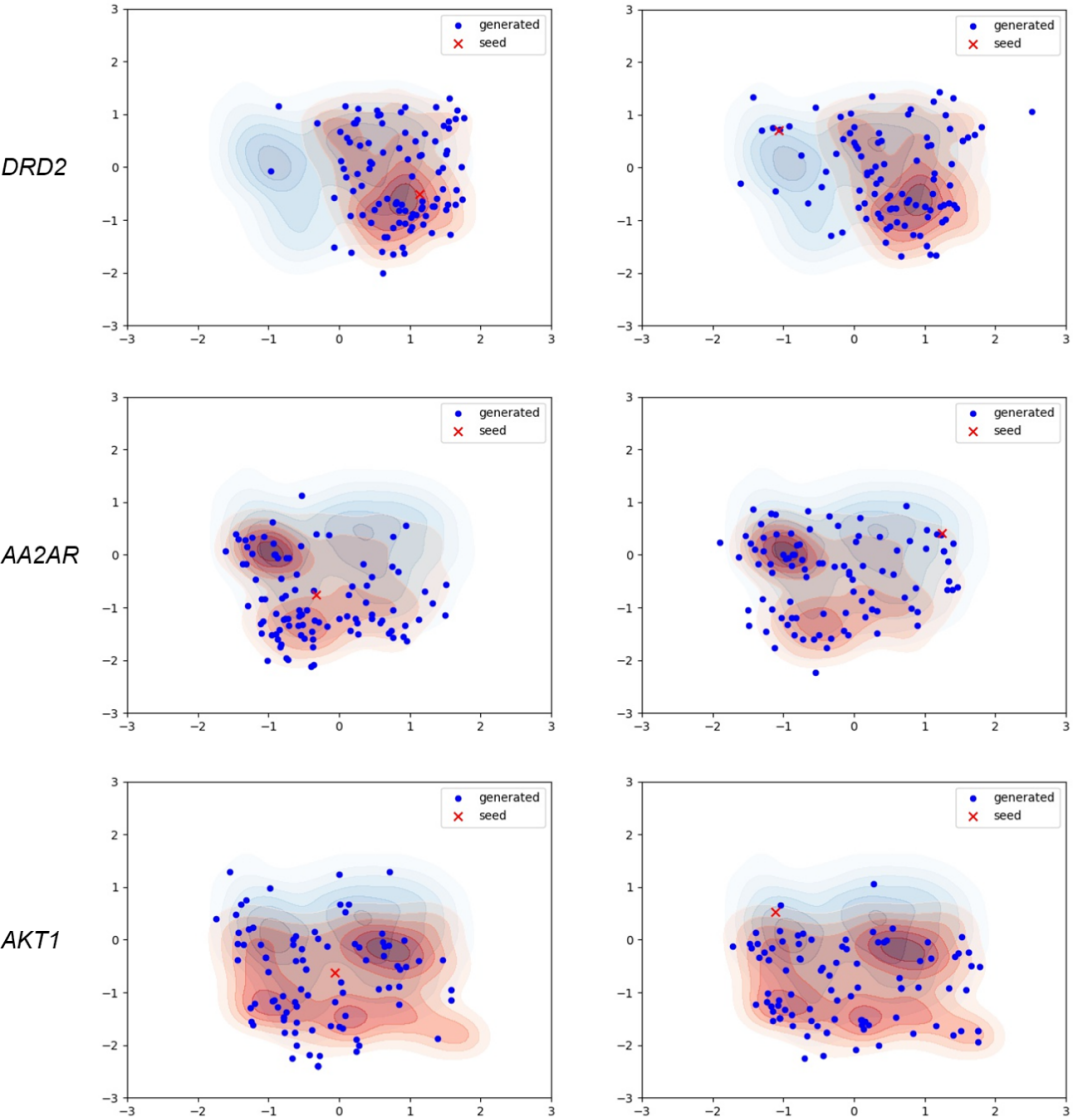
Distribution of compounds generated using IEV2Mol for each protein. The chemical space is visualized using principal component analysis (PCA) to reduce the dimensionality of the ECFP4 fingerprints (2048 bits) to two dimensions. The red crosses represent the seed compounds; the blue density map shows the distribution of 10,000 randomly selected compounds from the DM-QP-1 M data set, while the red density map shows the distribution of the active compound data set. The blue dots correspond to the compounds generated based on each seed compound, demonstrating the ability of the model to generate molecules that cover the chemical space of active compounds.

**Figure 5:**
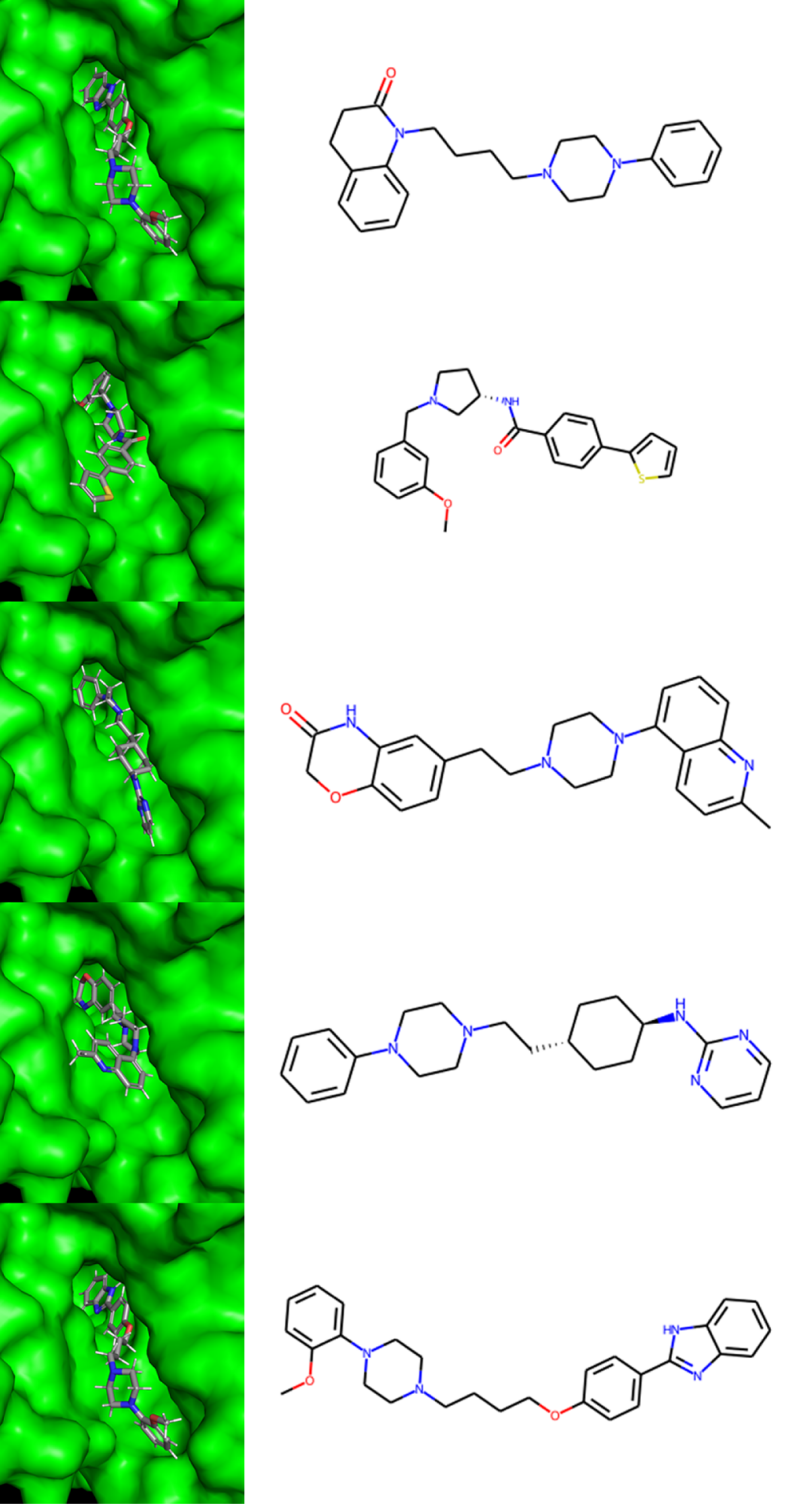
Docking poses generated by IEV2Mol for DRD2 using the Glide HTVS mode for the top four compounds with the highest IEV cosine similarity among those with Tanimoto coefficients less than 0.5. The seed compound is shown at the top, with the four generated compounds below it.

Figure 5 shows the docking poses of the seed compound (top) and the four compounds generated by IEV2Mol (bottom four). These generated compounds were selected from among compounds with Tanimoto coefficients less than 0.5 and with high IEV cosine similarity to the seed compound. The docking poses were obtained using the Glide HTVS mode, indicating the potential for IEV2Mol to generate structurally diverse compounds that may interact with its target protein, DRD2.

## Conclusion

In this study, we proposed a new VAE model, IEV2Mol, which generates compounds using information about the interaction between a compound and a protein, called the interaction energy vector (IEV). By using the IEVs calculated between the compound and the target protein as input, IEV2Mol generates compounds with similar IEVs for that target protein. This is accomplished by combining latent representations derived from IEVs between the seed compound and the target protein with latent representations randomly obtained from the vast chemical space and decoding them.

A series of experiments showed that compared to those of other generative models such as JTVAE and IFP-RNN, compounds generated with IEV2Mol tend to have IEVs similar to those of the seed compound, although they are structurally less similar to the seed compound. Although IEV2MOL has the limitation that it requires the tertiary structure of the target protein and known ligand data, it has the potential to be a useful tool in the hit discovery process because it can generate compounds with similar interactions, regardless of their structural similarity to the seed compound. For future work, to streamline compound generation targeting various proteins, it is conceivable to condition not only on vectors related to the interaction between proteins and small molecule compounds but also on fingerprints learned from the entire protein structure.^49−51^

## Data Availability

The source code and trained models for IEV2Mol, JT-VAE, and IFP-RNN designed for generating compounds active against the DRD2, AA2AR, and AKT1, as well as the data sets (DM-QP-1M, DRD2 Active, and ChEMBL33) utilized in this study, are released under the MIT License and available at https://github.com/sekijima-lab/IEV2Mol. This repository also includes scripts for plotting figures and calculating metrics to evaluate the performance of the different methods.

## Supporting Information Available

Chemical space visualization of seed compounds, active compounds, and generated compounds by IEV2Mol for each protein (PDF)
