# Supplementary material for: IEV2Mol: Molecular Generative Model Considering Protein–Ligand Interaction Energy Vectors
Source: J Chem Inf Model. 2024 Sep 10;64(18):6969–78. doi: 10.1021/acs.jcim.4c00842 (PMC11423338; doi:10.1021/acs.jcim.4c00842)
Supplement: Supplementary file 1 — ci4c00842_si_001.pdf [file ci4c00842_si_001.pdf]

Supporting Information for IEV2Mol: Molecular Generative Model Considering Protein-Ligand Interaction Energy Vectors

Mami Ozawa[1], Shogo Nakamura[2], Nobuaki Yasuo[3], and Masakazu Sekijima[*, 1]

[1]Department of Computer Science, Tokyo Institute of Technology, Kanagawa, Japan.
[2]Department of Life Science and Technology, Tokyo Institute of Technology, Kanagawa, Japan
[3]Academy for Convergence of Materials and Informatics (TAC-MI), Tokyo Institute of Technology, Tokyo, Japan.
[*]Email: sekijima@c.titech.ac.jp

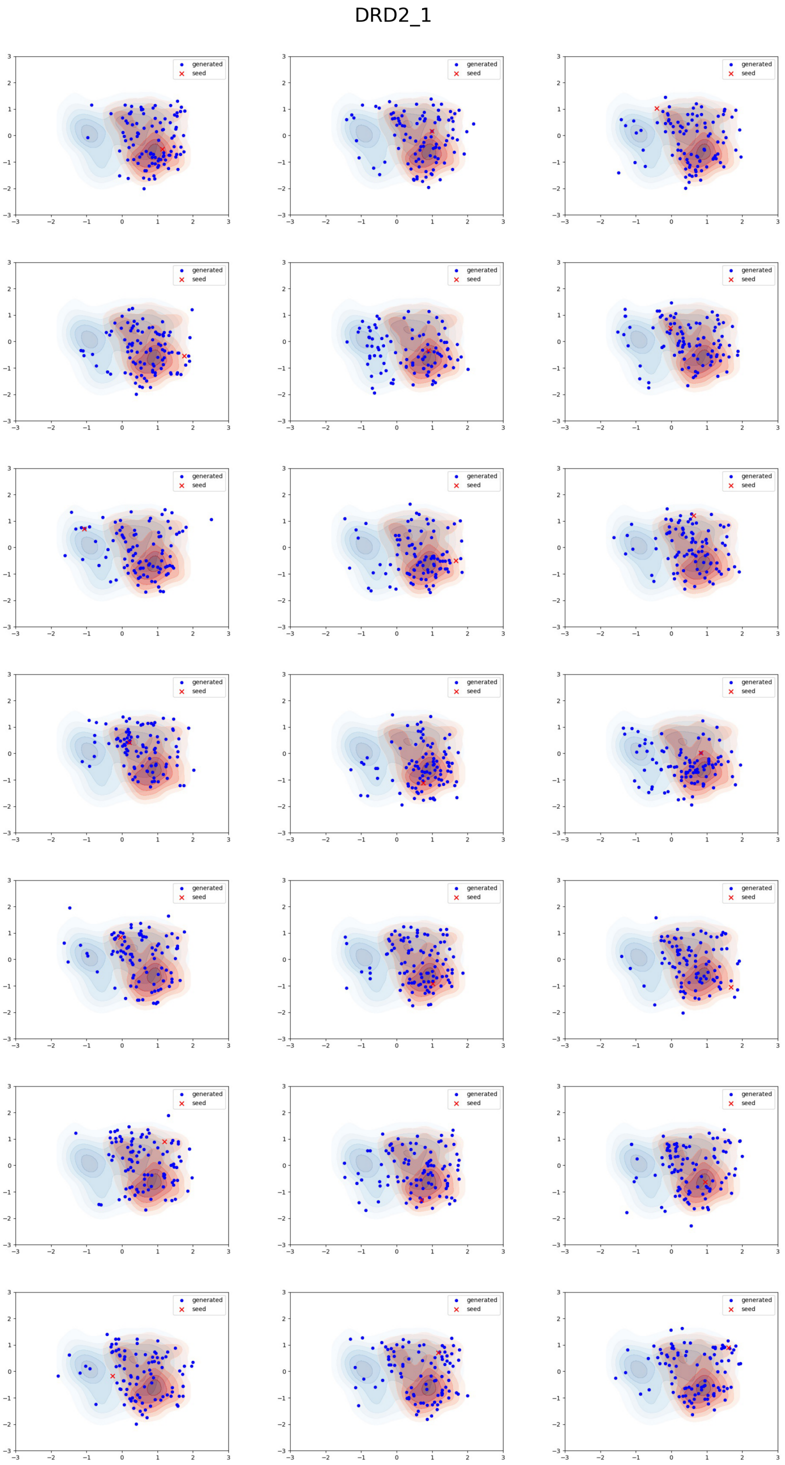

Figure S1: Distribution of compounds generated using IEV2Mol for DRD2. The chemical space is visualized using principal component analysis (PCA) to reduce the dimensionality of the ECFP4 fingerprints (2048 bits) to two dimensions. The red crosses represent the seed compounds; the blue density map shows the distribution of 10,000 randomly selected compounds from the DM-QP-1M dataset, while the red density map shows the distribution of the DRD2-Active dataset. The blue dots correspond to the compounds generated based on each seed compound, demonstrating the ability of the model to generate molecules that cover the chemical space of active compounds.

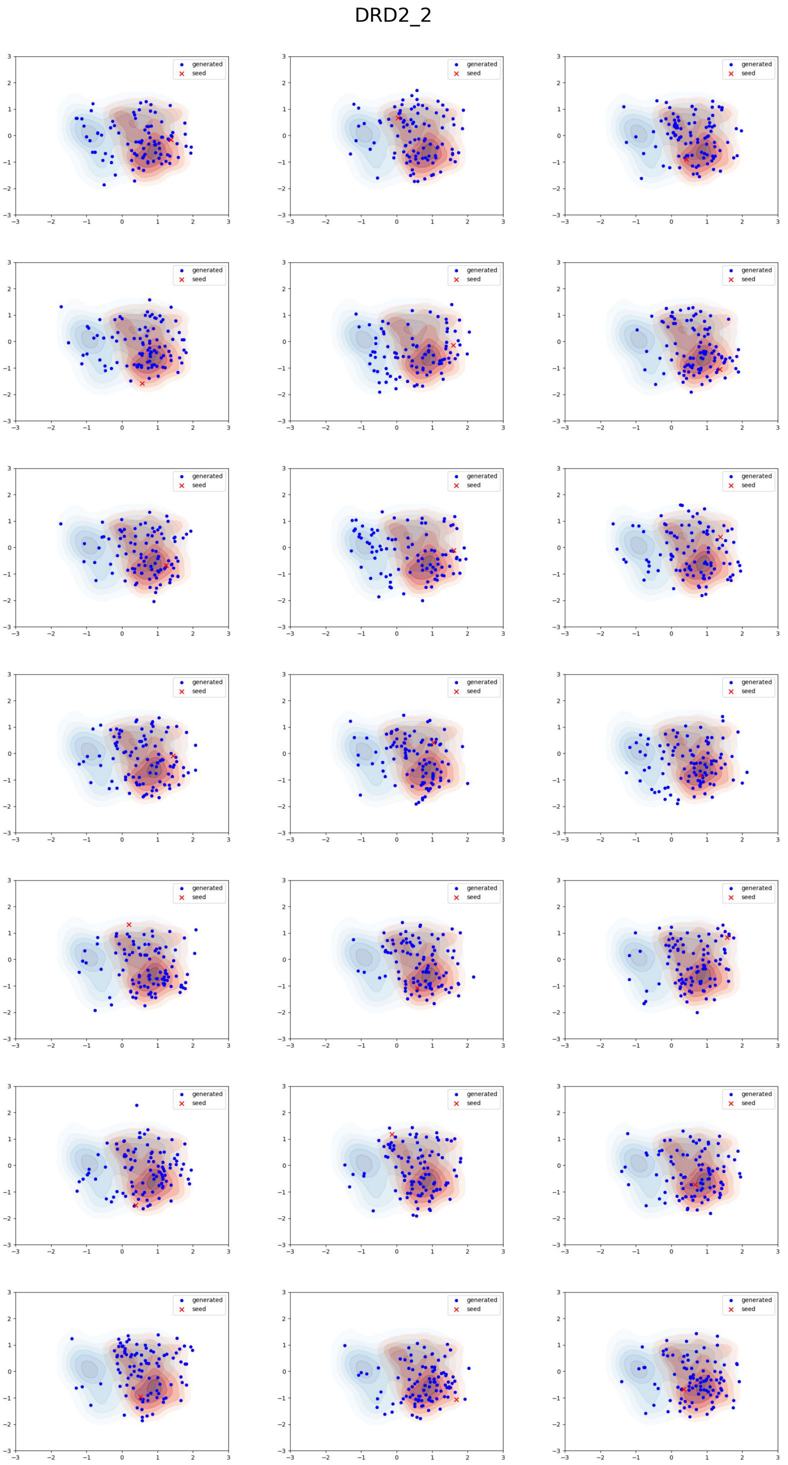

Figure S2: Distribution of compounds generated using IEV2Mol for DRD2. The chemical space is visualized using principal component analysis (PCA) to reduce the dimensionality of the ECFP4 fingerprints (2048 bits) to two dimensions. The red crosses represent the seed compounds; the blue density map shows the distribution of 10,000 randomly selected compounds from the DM-QP-1M dataset, while the red density map shows the distribution of the DRD2-Active dataset. The blue dots correspond to the compounds generated based on each seed compound, demonstrating the ability of the model to generate molecules that cover the chemical space of active compounds.

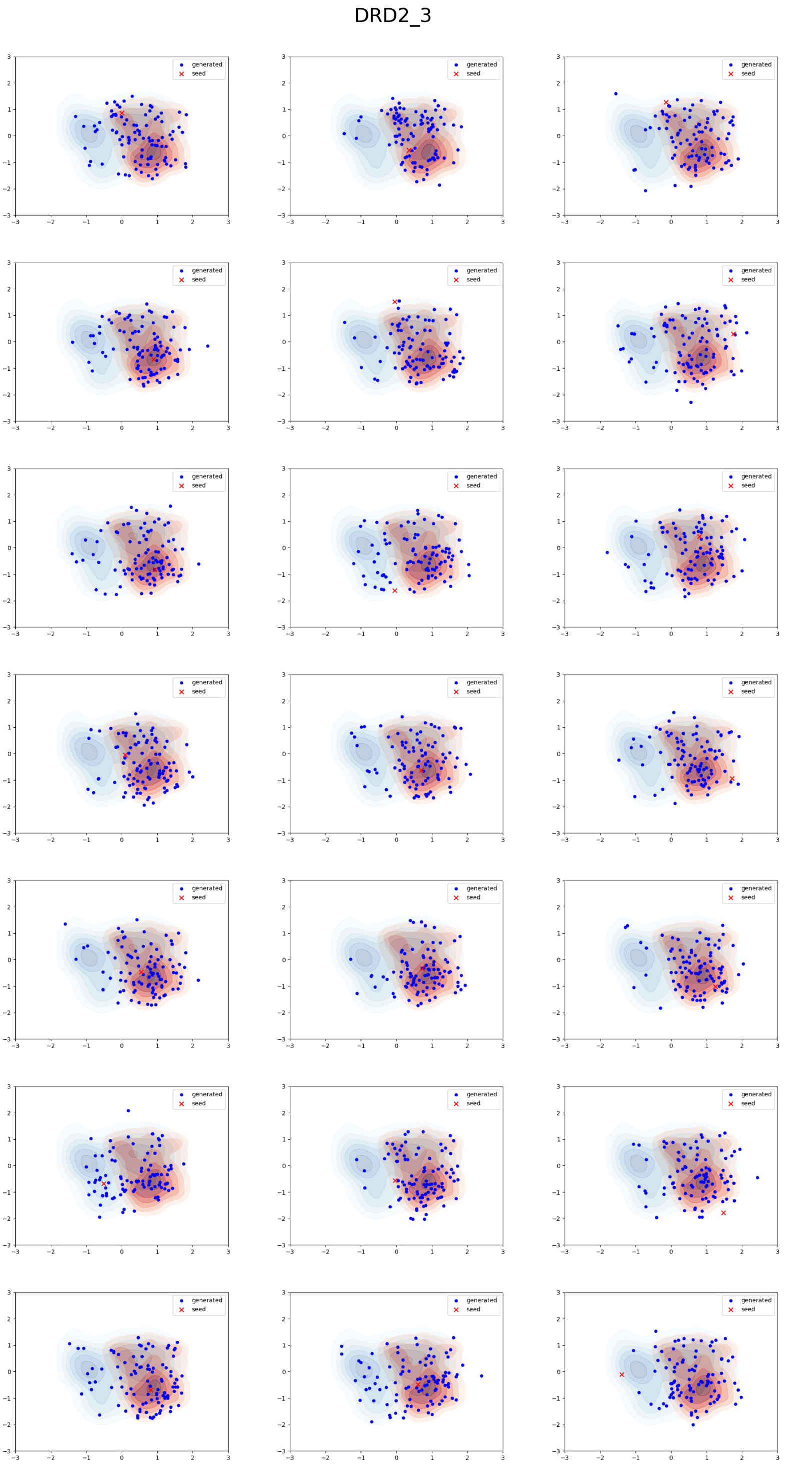

Figure S3: Distribution of compounds generated using IEV2Mol for DRD2. The chemical space is visualized using principal component analysis (PCA) to reduce the dimensionality of the ECFP4 fingerprints (2048 bits) to two dimensions. The red crosses represent the seed compounds; the blue density map shows the distribution of 10,000 randomly selected compounds from the DM-QP-1M dataset, while the red density map shows the distribution of the DRD2-Active dataset. The blue dots correspond to the compounds generated based on each seed compound, demonstrating the ability of the model to generate molecules that cover the chemical space of active compounds.

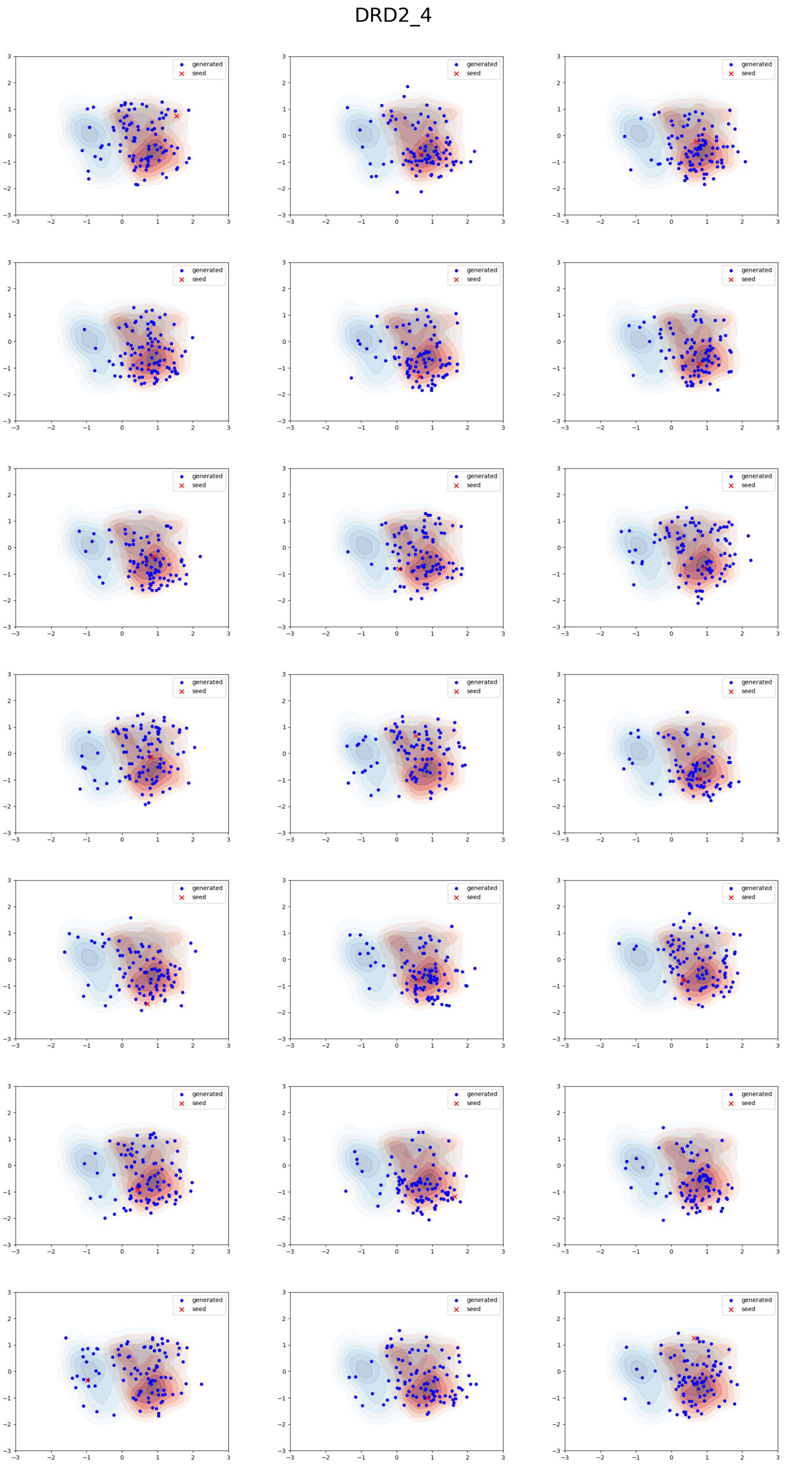

Figure S4: Distribution of compounds generated using IEV2Mol for DRD2. The chemical space is visualized using principal component analysis (PCA) to reduce the dimensionality of the ECFP4 fingerprints (2048 bits) to two dimensions. The red crosses represent the seed compounds; the blue density map shows the distribution of 10,000 randomly selected compounds from the DM-QP-1M dataset, while the red density map shows the distribution of the DRD2-Active dataset. The blue dots correspond to the compounds generated based on each seed compound, demonstrating the ability of the model to generate molecules that cover the chemical space of active compounds.

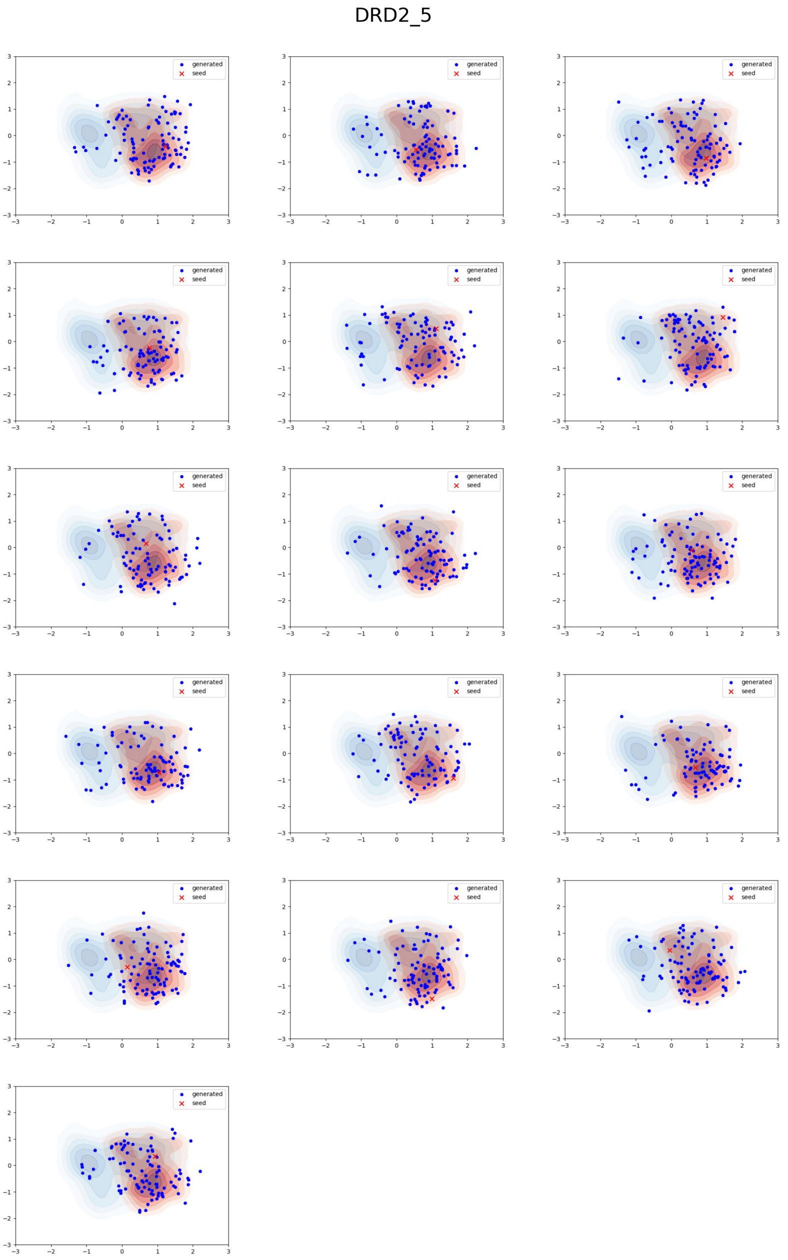

Figure S5: Distribution of compounds generated using IEV2Mol for DRD2. The chemical space is visualized using principal component analysis (PCA) to reduce the dimensionality of the ECFP4 fingerprints (2048 bits) to two dimensions. The red crosses represent the seed compounds; the blue density map shows the distribution of 10,000 randomly selected compounds from the DM-QP-1M dataset, while the red density map shows the distribution of the DRD2-Active dataset. The blue dots correspond to the compounds generated based on each seed compound, demonstrating the ability of the model to generate molecules that cover the chemical space of active compounds.

AA2AR_1

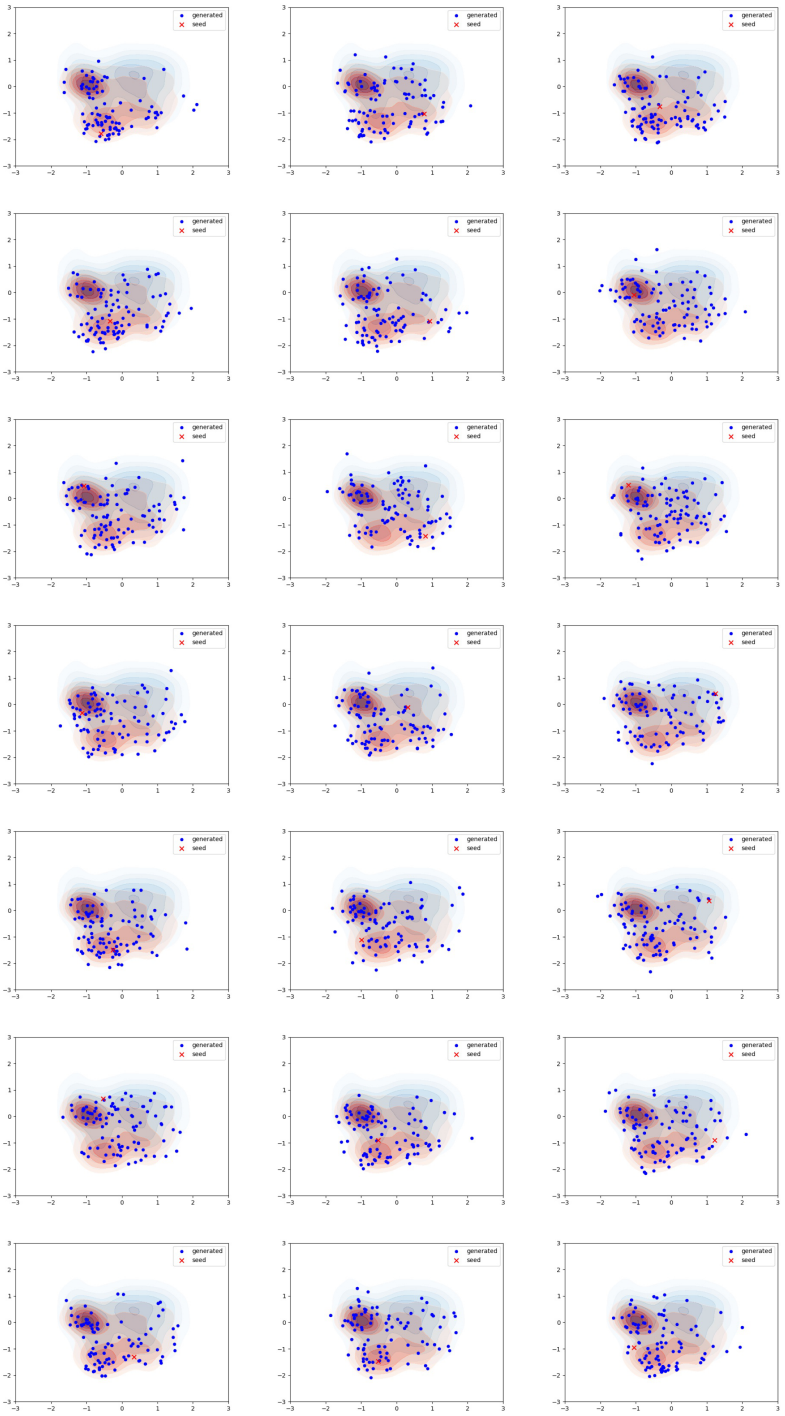

Figure S6: Distribution of compounds generated using IEV2Mol for AA2AR. The chemical space is visualized using principal component analysis (PCA) to reduce the dimensionality of the ECFP4 fingerprints (2048 bits) to two dimensions. The red crosses represent the seed compounds; the blue density map shows the distribution of 10,000 randomly selected compounds from the DM-QP-1M dataset, while the red density map shows the distribution of the DRD2-Active dataset. The blue dots correspond to the compounds generated based on each seed compound, demonstrating the ability of the model to generate molecules that cover the chemical space of active compounds.

AA2AR_2

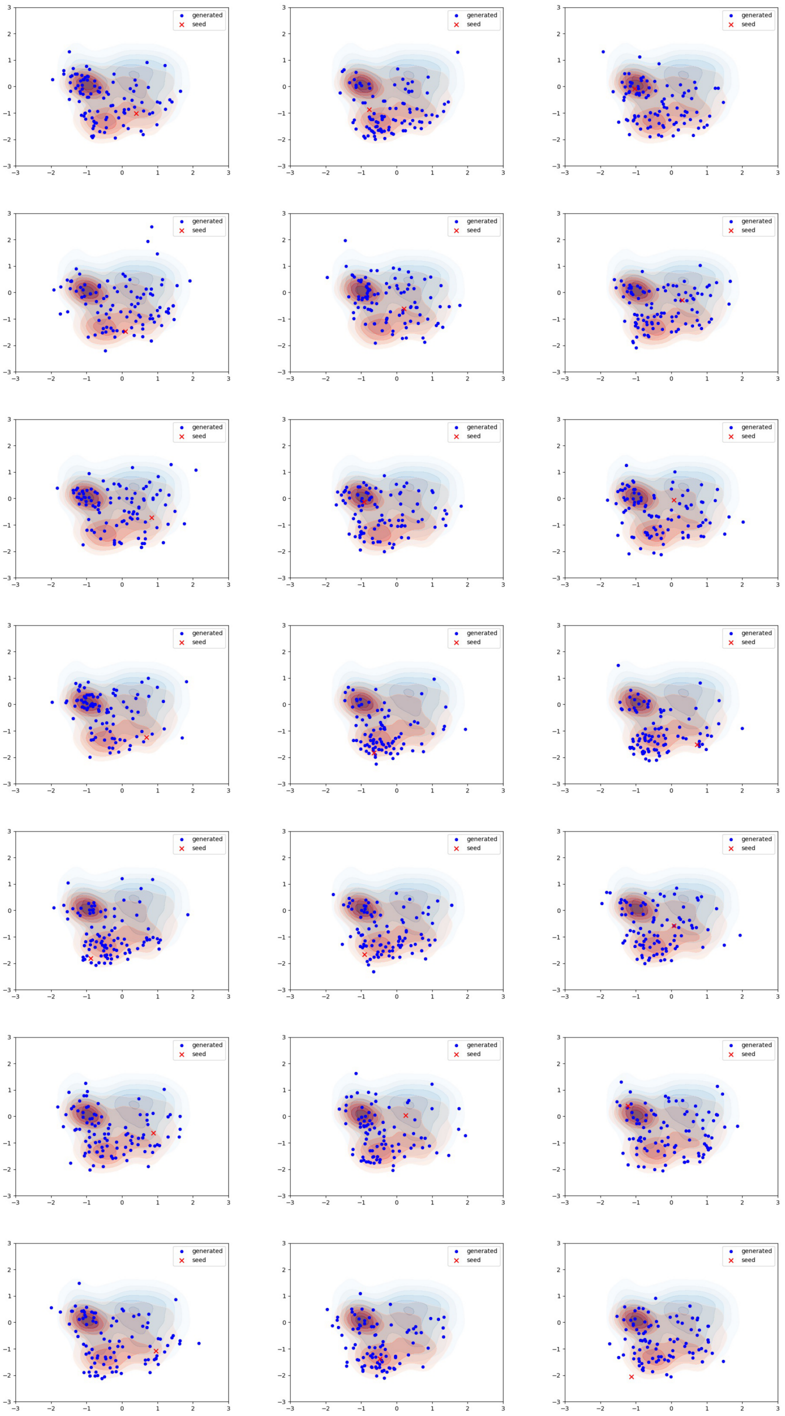

Figure S7: Distribution of compounds generated using IEV2Mol for AA2AR. The chemical space is visualized using principal component analysis (PCA) to reduce the dimensionality of the ECFP4 fingerprints (2048 bits) to two dimensions. The red crosses represent the seed compounds; the blue density map shows the distribution of 10,000 randomly selected compounds from the DM-QP-1M dataset, while the red density map shows the distribution of the DRD2-Active dataset. The blue dots correspond to the compounds generated based on each seed compound, demonstrating the ability of the model to generate molecules that cover the chemical space of active compounds.

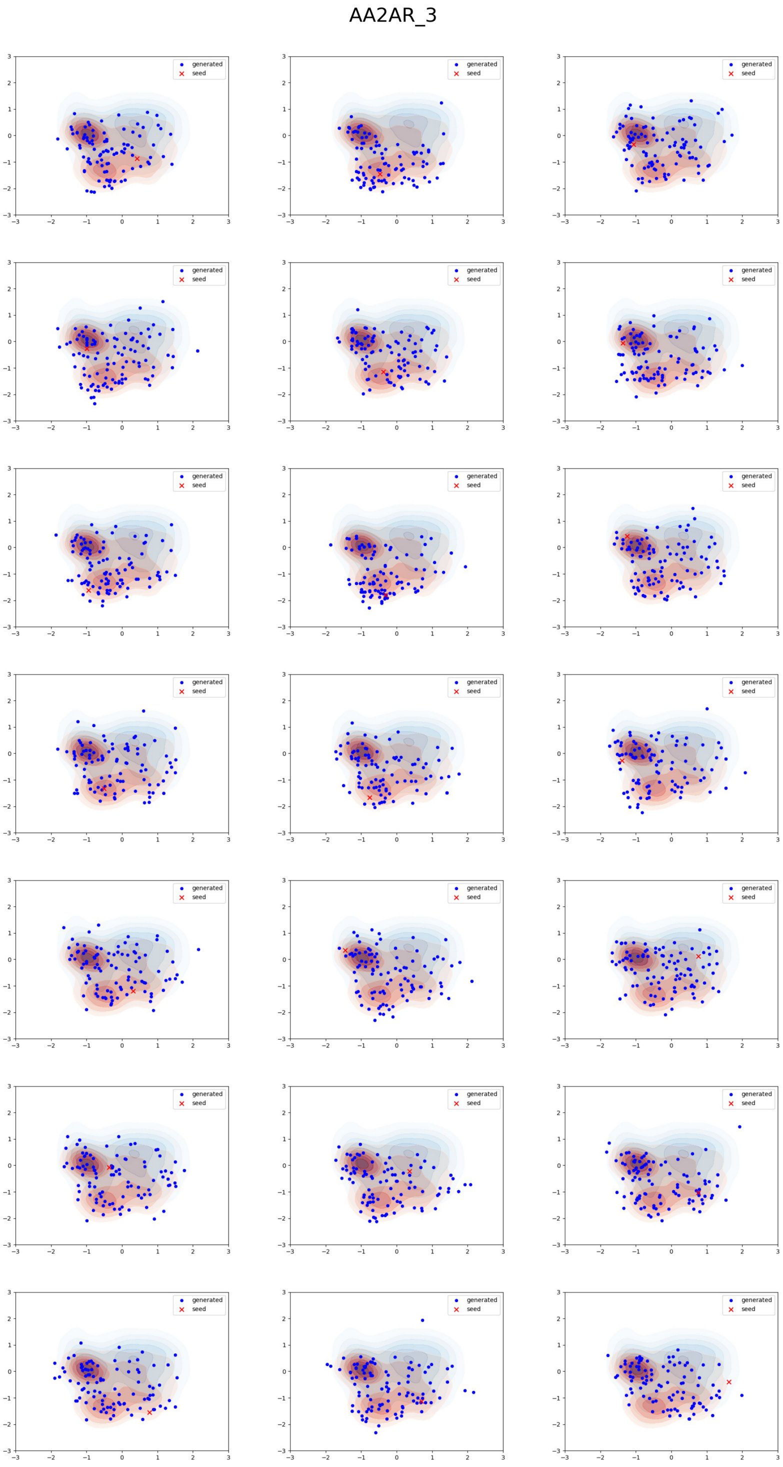

Figure S8: Distribution of compounds generated using IEV2Mol for AA2AR. The chemical space is visualized using principal component analysis (PCA) to reduce the dimensionality of the ECFP4 fingerprints (2048 bits) to two dimensions. The red crosses represent the seed compounds; the blue density map shows the distribution of 10,000 randomly selected compounds from the DM-QP-1M dataset, while the red density map shows the distribution of the DRD2-Active dataset. The blue dots correspond to the compounds generated based on each seed compound, demonstrating the ability of the model to generate molecules that cover the chemical space of active compounds.

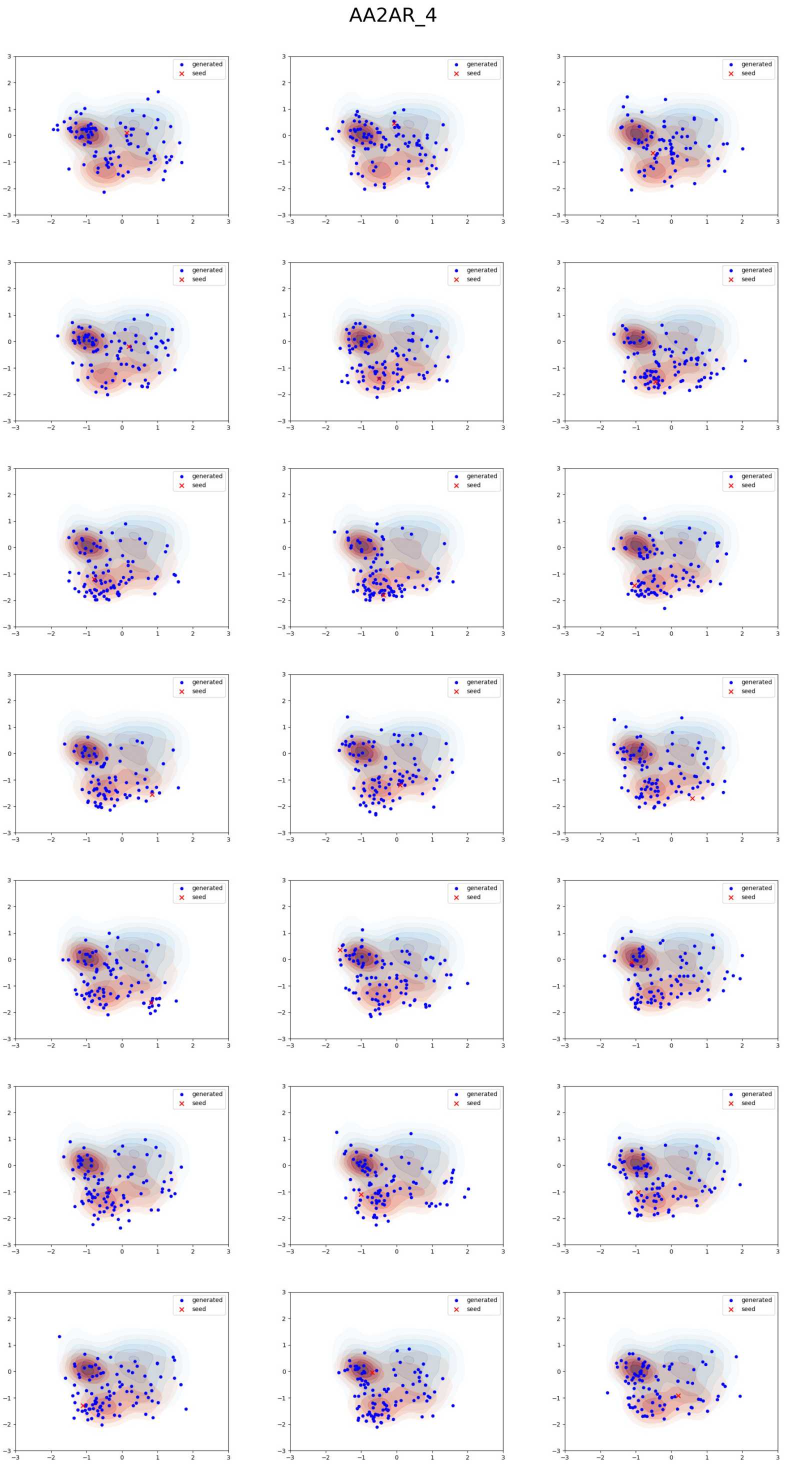

Figure S9: Distribution of compounds generated using IEV2Mol for AA2AR. The chemical space is visualized using principal component analysis (PCA) to reduce the dimensionality of the ECFP4 fingerprints (2048 bits) to two dimensions. The red crosses represent the seed compounds; the blue density map shows the distribution of 10,000 randomly selected compounds from the DM-QP-1M dataset, while the red density map shows the distribution of the DRD2-Active dataset. The blue dots correspond to the compounds generated based on each seed compound, demonstrating the ability of the model to generate molecules that cover the chemical space of active compounds.

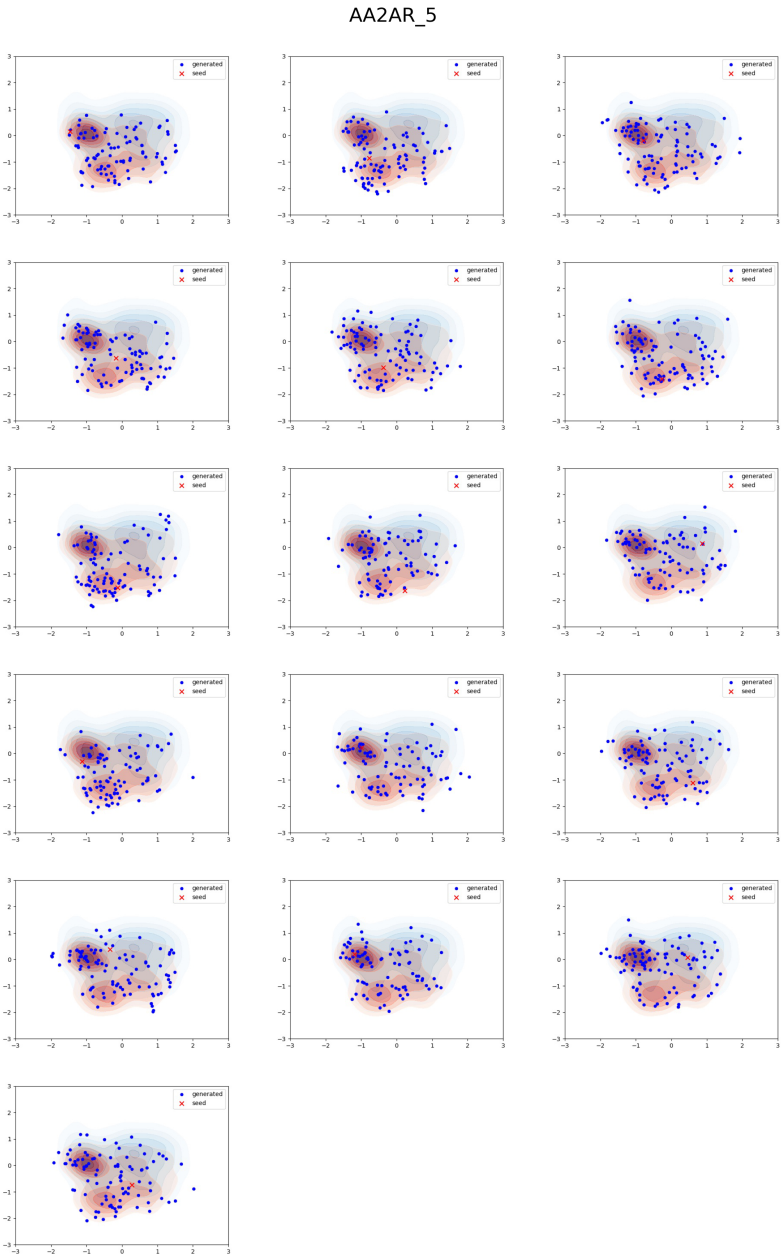

Figure S10: Distribution of compounds generated using IEV2Mol for AA2AR. The chemical space is visualized using principal component analysis (PCA) to reduce the dimensionality of the ECFP4 fingerprints (2048 bits) to two dimensions. The red crosses represent the seed compounds; the blue density map shows the distribution of 10,000 randomly selected compounds from the DM-QP-1M dataset, while the red density map shows the distribution of the DRD2-Active dataset. The blue dots correspond to the compounds generated based on each seed compound, demonstrating the ability of the model to generate molecules that cover the chemical space of active compounds.

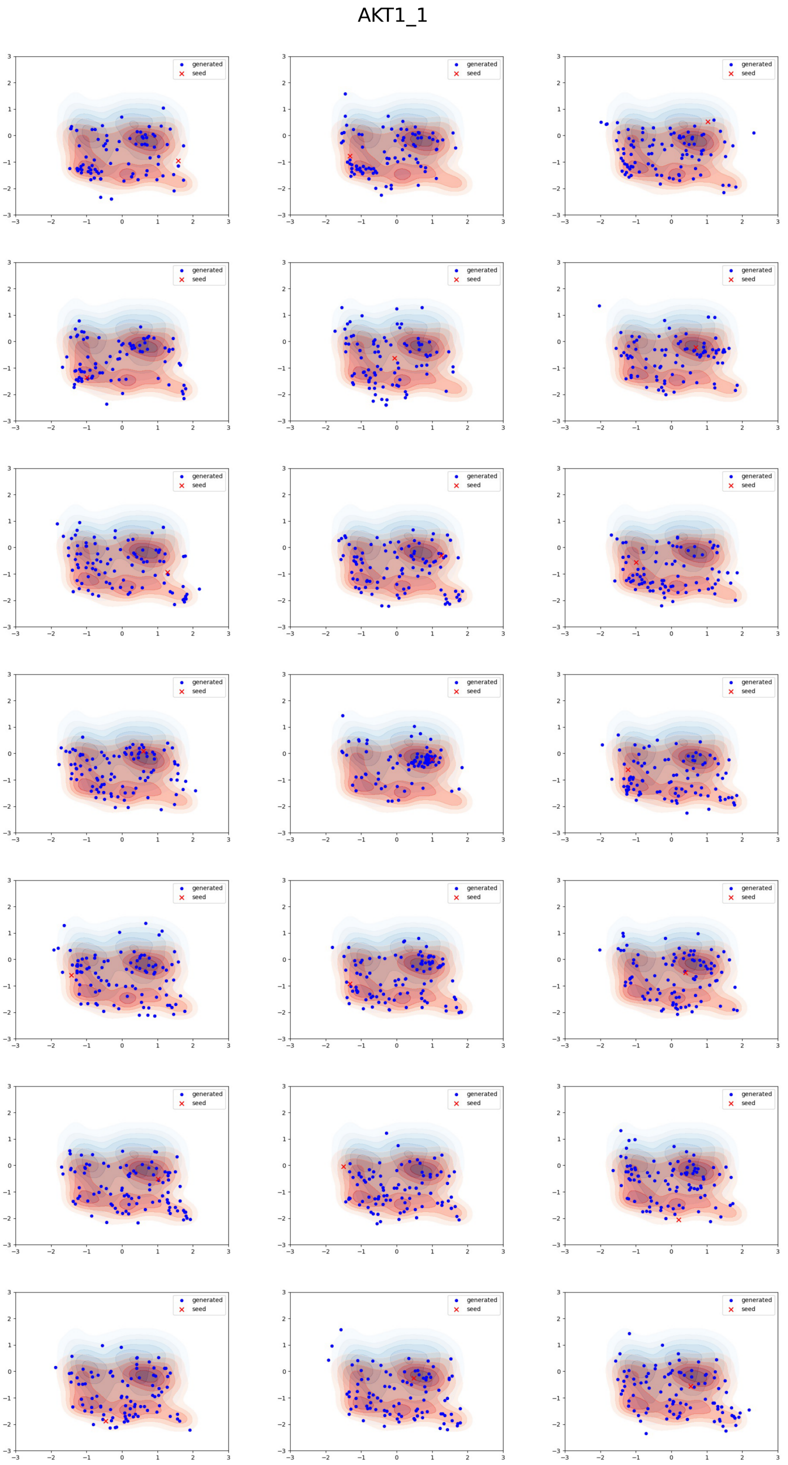

Figure S11: Distribution of compounds generated using IEV2Mol for AKT1. The chemical space is visualized using principal component analysis (PCA) to reduce the dimensionality of the ECFP4 fingerprints (2048 bits) to two dimensions. The red crosses represent the seed compounds; the blue density map shows the distribution of 10,000 randomly selected compounds from the DM-QP-1M dataset, while the red density map shows the distribution of the DRD2-Active dataset. The blue dots correspond to the compounds generated based on each seed compound, demonstrating the ability of the model to generate molecules that cover the chemical space of active compounds.

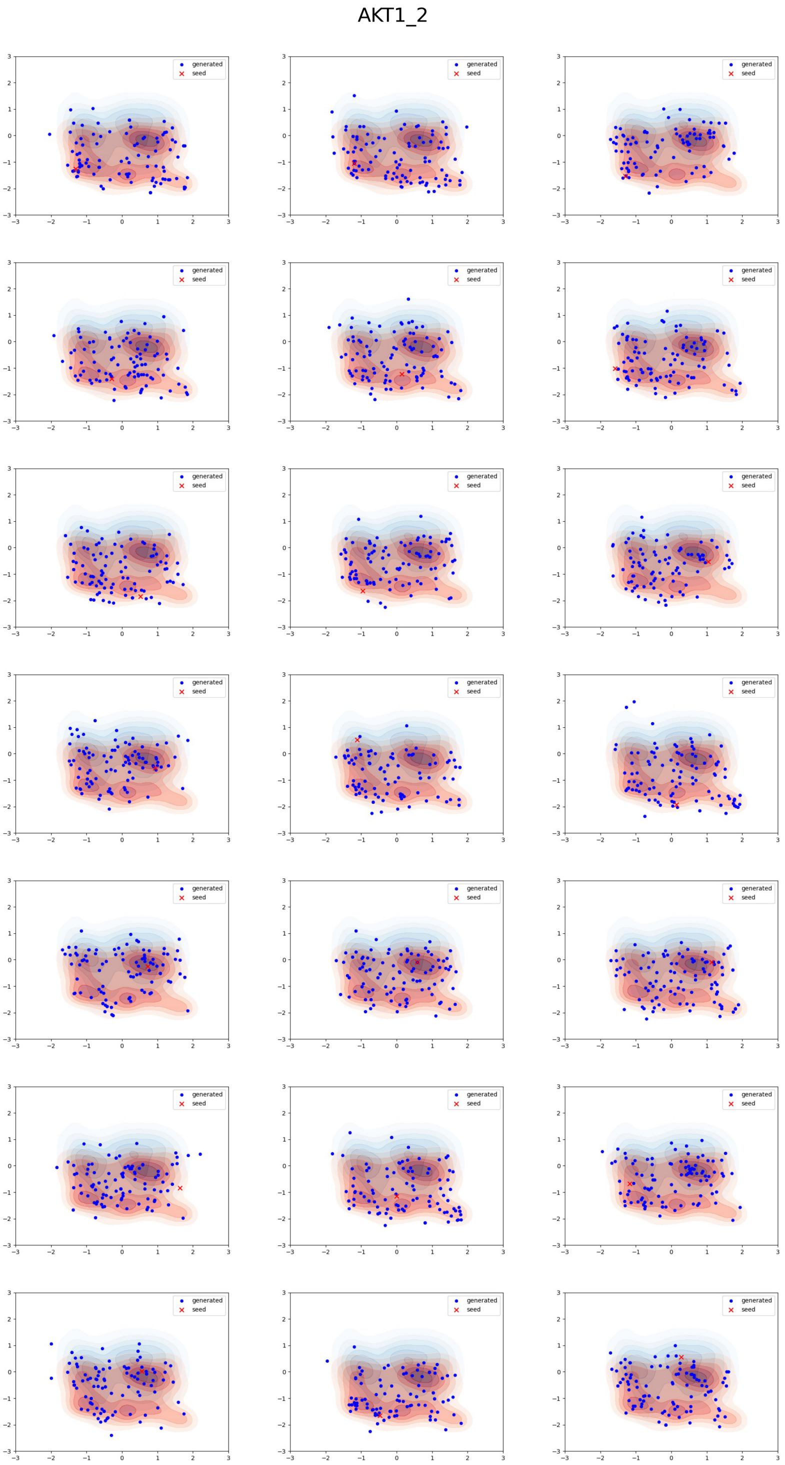

Figure S12: Distribution of compounds generated using IEV2Mol for AKT1. The chemical space is visualized using principal component analysis (PCA) to reduce the dimensionality of the ECFP4 fingerprints (2048 bits) to two dimensions. The red crosses represent the seed compounds; the blue density map shows the distribution of 10,000 randomly selected compounds from the DM-QP-1M dataset, while the red density map shows the distribution of the DRD2-Active dataset. The blue dots correspond to the compounds generated based on each seed compound, demonstrating the ability of the model to generate molecules that cover the chemical space of active compounds.

AKT1_3

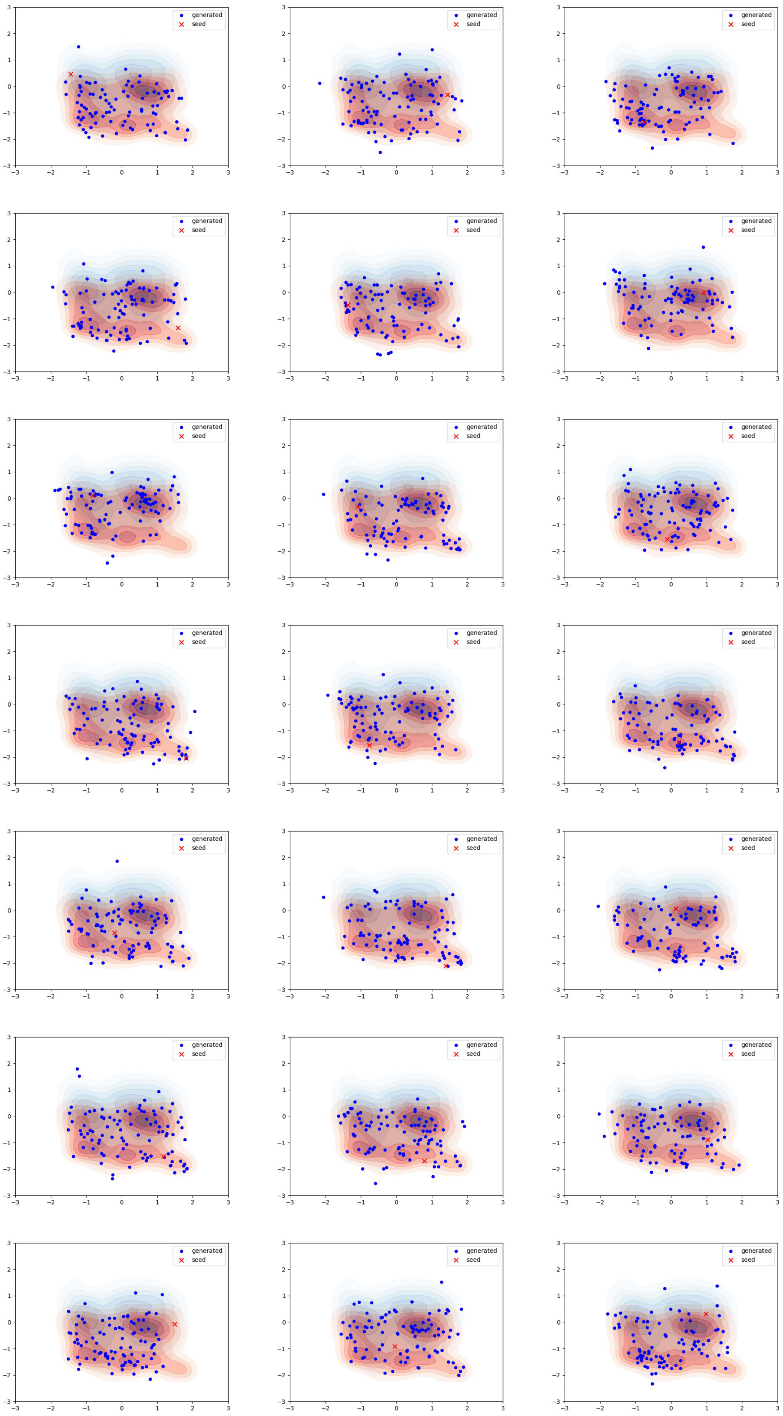

Figure S13: Distribution of compounds generated using IEV2Mol for AKT1. The chemical space is visualized using principal component analysis (PCA) to reduce the dimensionality of the ECFP4 fingerprints (2048 bits) to two dimensions. The red crosses represent the seed compounds; the blue density map shows the distribution of 10,000 randomly selected compounds from the DM-QP-1M dataset, while the red density map shows the distribution of the DRD2-Active dataset. The blue dots correspond to the compounds generated based on each seed compound, demonstrating the ability of the model to generate molecules that cover the chemical space of active compounds.

AKT1_4

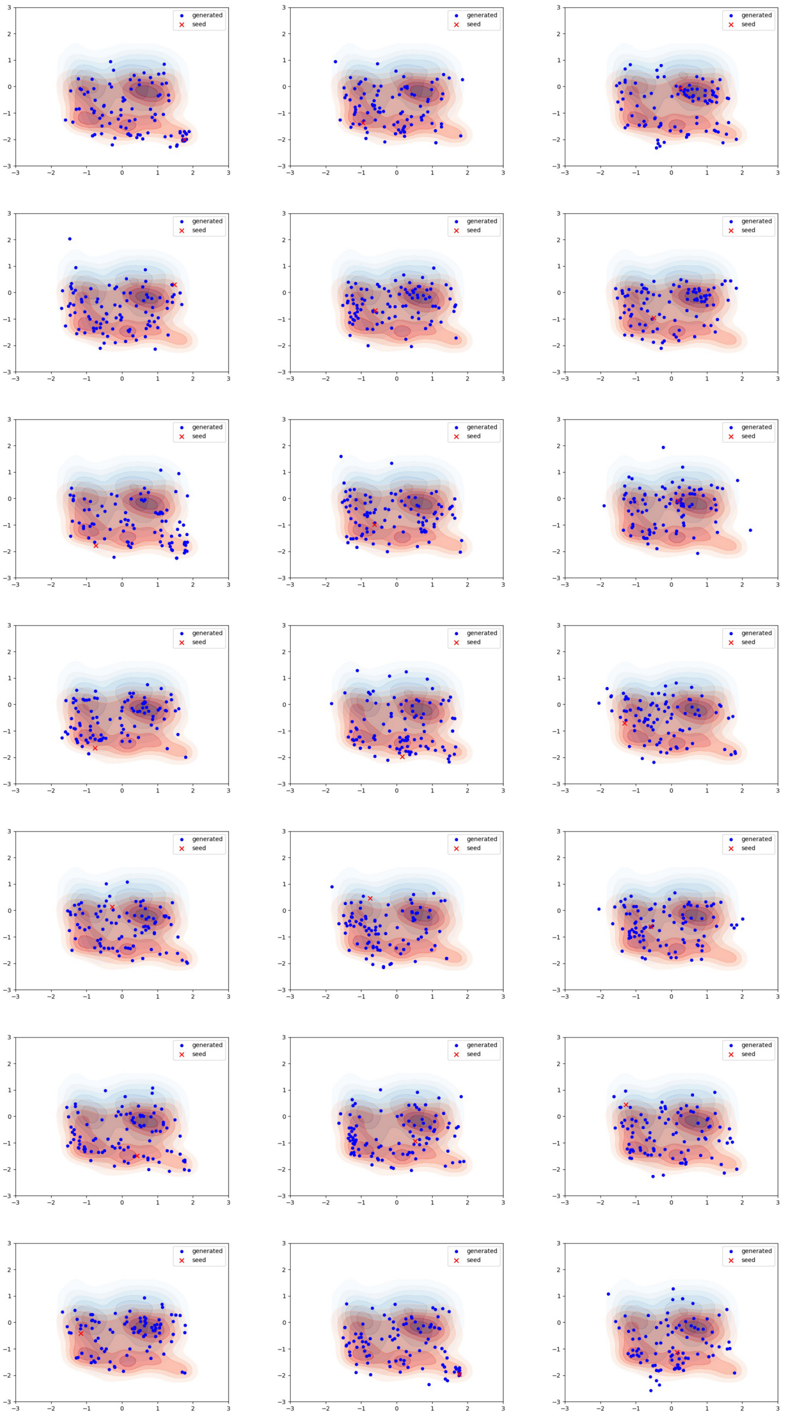

Figure S14: Distribution of compounds generated using IEV2Mol for AKT1. The chemical space is visualized using principal component analysis (PCA) to reduce the dimensionality of the ECFP4 fingerprints (2048 bits) to two dimensions. The red crosses represent the seed compounds; the blue density map shows the distribution of 10,000 randomly selected compounds from the DM-QP-1M dataset, while the red density map shows the distribution of the DRD2-Active dataset. The blue dots correspond to the compounds generated based on each seed compound, demonstrating the ability of the model to generate molecules that cover the chemical space of active compounds.

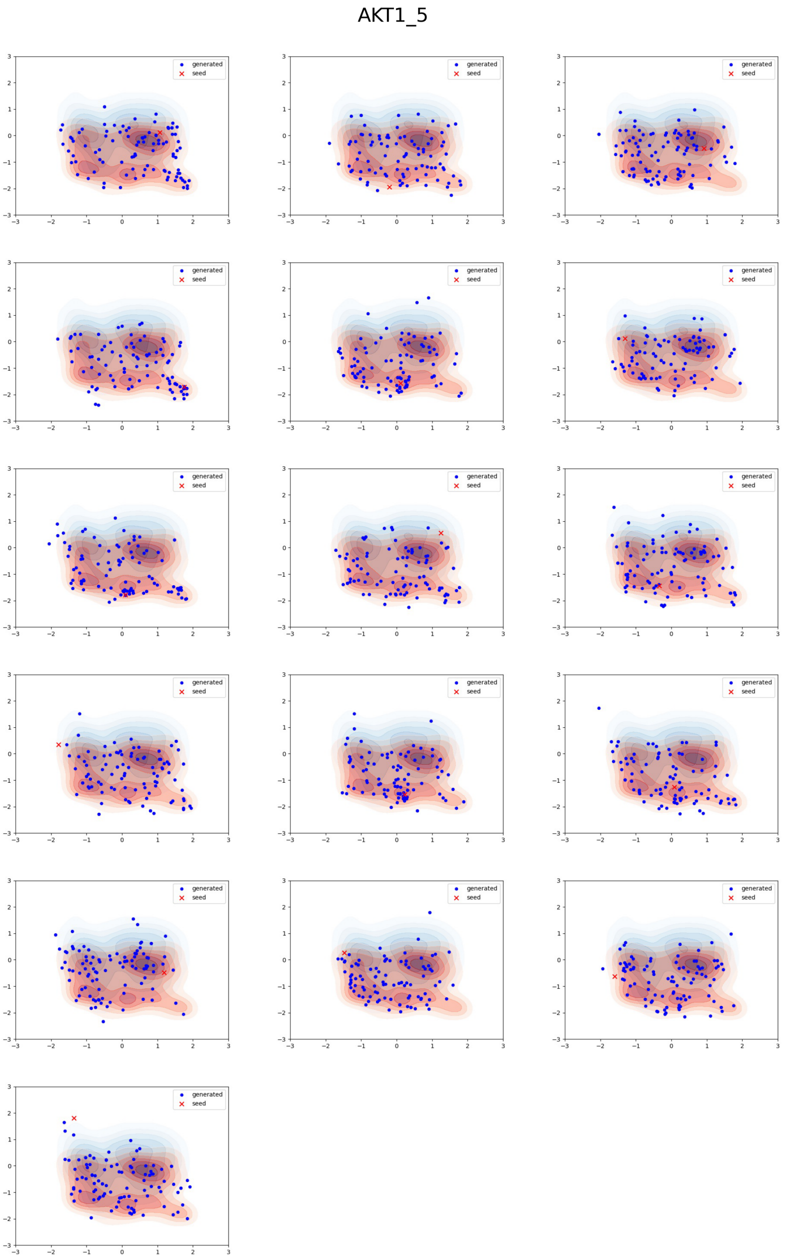

Figure S15: Distribution of compounds generated using IEV2Mol for AKT1. The chemical space is visualized using principal component analysis (PCA) to reduce the dimensionality of the ECFP4 fingerprints (2048 bits) to two dimensions. The red crosses represent the seed compounds; the blue density map shows the distribution of 10,000 randomly selected compounds from the DM-QP-1M dataset, while the red density map shows the distribution of the DRD2-Active dataset. The blue dots correspond to the compounds generated based on each seed compound, demonstrating the ability of the model to generate molecules that cover the chemical space of active compounds.
